# Comprehensive investigation into the influence of glycosylation on head and neck squamous cell carcinoma and development of a prognostic model for risk assessment and anticipating immunotherapy

**DOI:** 10.3389/fimmu.2024.1364082

**Published:** 2024-03-18

**Authors:** Heng Ma, Ludan Xiong, Bohui Zhao, Zhuledesi Hahan, Minghui Wei, Hengmei Shi, Susu Yang, Qianhe Ren

**Affiliations:** ^1^ Department of Head and Neck Surgery, National Cancer Center/National Clinical Research Center for Cancer/Cancer Hospital & Shenzhen Hospital, Chinese Academy of Medical Sciences and Peking Union Medical College, Shenzhen, China; ^2^ Department of GCP Center, National Cancer Center/National Clinical Research Center for Cancer/Cancer Hospital & Shenzhen Hospital, Chinese Academy of Medical Sciences and Peking Union Medical College, Shenzhen, China; ^3^ Department of Obstetrics and Gynecology, Women’s Hospital of Nanjing Medical University, Nanjing Maternity and Child Health Care Hospital, Nanjing, China; ^4^ Department of Breast Surgery, The First Affiliated Hospital of Nanjing Medical University, Nanjing, China; ^5^ Department of Thoracic Surgery, The First Affiliated Hospital of Nanjing Medical University, Nanjing, China

**Keywords:** head and neck squamous cell carcinoma, glycosylation, methylation, prognosis, tumor immune microenvironment, immunotherapy

## Abstract

**Background:**

It has been well established that glycosylation plays a pivotal role in initiation, progression, and therapy resistance of several cancers. However, the correlations between glycosylation and head and neck squamous cell carcinoma (HNSCC) have not been elucidated in detail.

**Methods:**

The paramount genes governing glycosylation were discerned via the utilization of the Protein-Protein Interaction (PPI) network and correlation analysis, coupled with single-cell RNA sequencing (scRNA-seq) analysis. To construct risk models exhibiting heightened predictive efficacy, cox- and lasso-regression methodologies were employed, and the veracity of these models was substantiated across both internal and external datasets. Subsequently, an exploration into the distinctions within the tumor microenvironment (TME), immunotherapy responses, and enriched pathways among disparate risk cohorts ensued. Ultimately, cell experiments were conducted to validate the consequential impact of SMS in Head and Neck Squamous Cell Carcinoma (HNSCC).

**Results:**

A total of 184 genes orchestrating glycosylation were delineated for subsequent scrutiny. Employing cox- and lasso-regression methodologies, we fashioned a 3-gene signature, proficient in prognosticating the outcomes for patients afflicted with HNSCC. Noteworthy observations encompassed distinctions in the Tumor Microenvironment (TME), levels of immune cell infiltration, and the presence of immune checkpoint markers among divergent risk cohorts, holding potentially consequential implications for the clinical management of HNSCC patients.

**Conclusion:**

The prognosis of HNSCC can be proficiently anticipated through risk signatures based on Glycosylation-related genes (GRGs). A thorough delineation of the GRGs signature in HNSCC holds the potential to facilitate the interpretation of HNSCC’s responsiveness to immunotherapy and provide innovative strategies for cancer treatment.

## 1 Introduction

Head and neck squamous cell carcinomas (HNSCCs) emanate from the mucosal epithelium within the oral cavity, pharynx, and larynx, constituting the most prevalent malignancies manifesting within the cephalic and cervical regions (1). As an extremely malignant tumor, HNSCC stands as the sixth most prevalent neoplastic condition globally, registering 890,000 newly diagnosed cases and 450,000 fatalities in the year 2018 (2). The frequency of HNSCC persistently ascends and is prognosticated to escalate by 30%, projecting an annual occurrence of 1.08 million new cases by the year 2030 (3). The primary modalities of therapeutic intervention for locally or locoregionally confined HNSCC encompass resection, radiation, and systemic therapy. Surgical procedures are frequently chosen for malignancies within the oral cavity, whereas radiation is more commonly employed for pharyngeal and laryngeal cancers. In the case of laryngeal cancers, a moderately hypo-fractionated radiation schedule yields superior locoregional control and survival outcomes compared to standard radiation therapy (4, 5). Progress in minimally invasive resection techniques, such as transoral robotic or laser resection, and larynx-preserving partial laryngectomy, alongside advancements in reconstructive methodologies, have broadened the indications for primary surgical management under the expertise of high-volume head and neck surgical oncologists (6). Even though, most HNSCC patients still have to encounter unfavorable prognosis due to the lack of early diagnosis and precise treatment. Therefore, it is urgent to develop appropriate methods applied for diagnosis and therapy of HNSCC.

Glycosylation, a fundamental and prominent post-translational modification of proteins, plays pivotal roles in diverse cellular processes, including cell proliferation, differentiation, oncogenic transformation, adhesion, and immune surveillance of cancers (7, 8). Recent attention has focused on changes in cellular glycosylation as a key component of neoplastic progression (9). Nowadays, state-of-the-art technologies have provided novel opportunities and methodologies for scrutinizing cancer-associated glycosylation. Consequently, glycans, along with anomalously glycosylated proteinaceous cancer biomarkers, have gained escalating recognition (10). Glycans intricately participate in foundational molecular and cellular processes inherent to cancer, encompassing cell signaling and intercellular communication, disintegration and infiltration of neoplastic cells, interactions between cells and the extracellular matrix, tumor angiogenesis, immune modulation, and the establishment of metastatic lesions. The significance of glycans in cancer is underscored by the observation that modifications in glycosylation intricately govern the evolution and advancement of cancer, functioning as pivotal biomarkers and furnishing a repertoire of precise targets for therapeutic intervention (11). Cancer immunotherapy employing immune checkpoint blockade (ICB), encompassing antibodies that impede cytotoxic T lymphocyte protein 4 (CTLA-4) and programmed cell death protein 1 [PD-(L)1], has enhanced the outcomes for cancer patients. However, the benefits of the presently available ICB are realized by only a minority of individuals (12). Ongoing investigations explore novel target pathways, and combinatory strategies involving agents obstructing both CTLA-4 and PD-(L)1 exhibit encouraging preclinical and early clinical efficacy (13). The upregulation of sialic acid–containing glycans in the tumor microenvironment, referred to as tumor hypersialylation, contributes to the establishment of an immunosuppressive milieu and attenuates antitumor immune responses by engaging immunomodulatory Siglecs expressed on tumor-infiltrating immune cells (14, 15). Recent research indicates the sialoglycan-Siglec axis as a novel immune checkpoint that can be addressed to stimulate innate and adaptive antitumor immunity (16). Nevertheless, in light of the presence of numerous Siglecs and their extensive array of expression within the immune system, the precise mechanism remains ambiguous. The complexity of glycan structures and their interactions with Siglecs adds a layer of intricacy to our comprehension. While we have made strides in elucidating the roles of specific sialoglycans and Siglecs in modulating immune responses, there are still gaps in our understanding of the precise mechanisms underlying these interactions, particularly in diverse physiological contexts (17). While our knowledge of the sialoglycan-Siglec axis has expanded, there are still uncertainties and challenges that necessitate further research. Acknowledging these limitations not only enhances the credibility of our findings but also guides future investigations aimed at unraveling the complexities of glycan-mediated immune regulation.

In this investigation, we acquired single-cell RNA sequencing (scRNA-seq) data and transcriptome data from accessible databases to discriminate glycosylation and construct a risk signature based on Glycosylation-Related Genes (GRGs) for Head and Neck Squamous Cell Carcinoma (HNSCC). We assessed the clinical import of the GRGs-based signature and subsequently scrutinized the immune milieu and responsiveness to immunotherapy correlated with it. Vitro experiments were used to validate the crucial impacts core gene has on HNSCC cell lines. With the findings, early modalities are more likely to be applied for those patients diagnosed with HNSCC, who could have more favorable outcomes.

## 2 Methods

### 2.1 Data collection and processing

HNSCC single-cell RNA sequencing (scRNA-seq) data were procured from the GEO database [Home - GEO - NCBI (nih.gov)] under accession number GSE234933, 14 samples of which were selected for our research, comprising of 7 primary malignant tumor samples and 7 metastatic ones. We systematically excluded individual cells exhibiting expression of fewer than three genes or manifesting expression in fewer than 250 genes. Subsequently, the proportion of ribosomal RNA (rRNA) and mitochondrial content was computed utilizing the PercentageFeatureSet function within the Seurat R package. As a result, a total of 28736 cells were acquired for subsequent analytical endeavors. Besides, we systematically gathered transcriptomic data and associated clinical information pertaining to HNSCC from TCGA repository [GDC (cancer.gov)]. Samples devoid of outcome status or survival-related data were omitted, resulting in the acquisition of 128 HNSCC specimens for external validation. The training cohort, comprised of 109 HNSCC samples, was derived from the GSE27020 dataset after discarding samples lacking follow-up information, retrieved from the Gene Expression Omnibus (GEO) database. The last, 184 glycosylation-related genes were obtained from GSEA (GSEA (gsea-msigdb.org)).

### 2.2 Pathway and enrichment analysis

In order to evaluate the functionalities of GRGs, gene set enrichment analysis (GSEA) was conducted utilizing the R packages ‘clusterProfiler’ and ‘limma,’ incorporating the hallmark gene sets (h.all.v7.5.symbols.gmt) and the Gene Ontology-Biological Processes (GO-BP) subsets derived from the canonical pathway gene sets (c2.cp.go.v7.5.symbols.gmt) (18).

In order to interrogate the profound biological processes associated with these disparately expressed GRGs, pathway and enrichment analyses were executed utilizing the R package ‘clusterProfiler.’ Gene Ontology (GO) and Kyoto Encyclopedia of Genes and Genomes (KEGG) enrichment analyses were conducted. P-adjusted values < 0.05 were deemed as significant thresholds.

### 2.3 Protein-protein interaction

To explore the protein-protein interaction in glycosylation-related genes, PPI network was constructed. Proteins with strong interactions were obtained from ‘STRING’ with interaction score was set as highest confidence (0.900). The Cytoscape software (version 3.9.0; https://cytoscape.org/) was utilized to build the PPI network. The cytoHubba plug-in (version 0.1; https://apps.cytoscape.org/apps/cytohubba) To analyze the network topology properties of the nodes, the CytoNCA plug-in (version 2.1.6; https://apps.cytoscape.org/apps/cytonca) was used, and the parameter was set to “without weight”. The crucial nodes of PPI network were obtained after arranging each node with the order of score.

### 2.4 Cell-type clustering and GRGs expression in scRNA

The scRNA-seq data pertaining to Head and Neck Squamous Cell Carcinoma (HNSCC) underwent re-analysis through the Seurat package (19). The primary objective was to systematically delineate the GRGs signature. Commencing the data preprocessing, cells expressing fewer than 250 or more than 6000 genes were excluded. The remaining expressed genes underwent log-normalization. Subsequently, the FindIntegrationAnchors function was employed. In order to diminish the data’s dimensionality, the t-distributed Stochastic Neighbor Embedding (tSNE) method was applied with a resolution of 0.1, utilizing 30 principal components. It is noteworthy that the tSNE method employed manifested non-linear characteristics. To categorize individual cells into distinct subgroups, the FindNeighbors and FindClusters functions were utilized, with a dimensionality of 30 and a resolution of 0.1. Thus, 18 cells clusters were obtained comprising of 28736 cells. The cellular entities were subsequently classified into eight principal cell types, guided by the identification of canonical marker genes. Noteworthy classifications include Epithelial cells, characterized by the presence of CDKN2A, CDH1, EPCAM, MUC5B, WFDC2, and PTPRT. T cells were discerned through the expression of CD247, CD2, and CD3E, while B cells were identified based on the manifestation of CD79A, CD79B, and MS4A1. Fibroblasts were distinguished by the expression of COL1A1, COL1A2, LUM, and DCN, and endothelial cells were marked by PECAM1, ENG, PLVAP, and CDH5. Cancer stem cells exhibited characteristic gene expression including EPCAM, CD24, SOX4, and KRT18. Additionally, Monocytes were identified by the presence of CST3 and LYZ, whereas Macrophages were marked by CD68, CD163, and CD14. Those marker genes were identified to play pivotal roles in specific cellular functions.

### 2.5 Trajectory analysis

Single-cell trajectory analysis was performed utilizing monocle 2 (v2.22.0). The count matrix derived from a specific cell type was employed, and genes expressed in fewer than 10 cells were preserved for subsequent analysis. The differentially expressed genes between High B4GALT1 fibroblasts and Low B4GALT1 fibroblasts (logFCfilter=1 adjPvalFilter=0.05) were selected for further analysis. For High B4GALT1 fibroblasts or Low B4GALT1 fibroblasts, the determination of ordering genes was predicated on their dispersion and expression levels across all genes. Ultimately, the trajectory was delineated through the reduceDimension function, employing the DDRTree method.

### 2.6 Cell–cell communication analysis

To comprehend the intricate communication network within the eight major cell types, an analysis of cell–cell communication was systematically undertaken through CellphoneDB (v3.1.0). Only ligand-receptor pairs with a P-value below 0.05 were deemed significant and retained for further investigation (The criteria about how the ligand-receptor pairs were identified as significant in CellphoneDB analysis were referred to database cellphonedb.org/ppi-resources). In the context of Cellchat (v1.4.0) analysis, all the ligand-receptor pairs, encompassing Secreted Signaling, ECM-Receptor, and Cell–Cell Contact categories, were employed to construct a comprehensive cell–cell communication network. This network was delineated across various cell types in primary malignant samples and metastasis, utilizing default parameters.

### 2.7 DNA methylation analysis

Data of DNA methylation in HNSCC was downloaded and further processed from GEO database (GSE178218). For methylated data, the beta value undergoes conversion to M value through the formula M = log2[beta/(1 - beta)] (20). The utmost methylation locus situated within the corresponding gene promoter region, encompassing TSS1500, TSS200’s, and 5’UTR, was employed to represent the methylation level of the gene promoter region (21). Subsequently, the R package limma was employed for the scrutiny and characterization of differentially expressed genes (DEGs) and differentially methylated CpG sites (DMCs) across tumor and normal samples. The Benjamini–Hochberg (BH) false discovery rate (FDR) method was applied to ascertain the adjusted p-value for each CpG site and gene. An FDR-adjusted p-value less than 0.05 served as the threshold criterion for the identification of DEGs and DMCs.

### 2.8 Construction of GRGs risk signature

The Differentially Expressed Genes (DEGs) exhibiting elevated expression levels in High B4GALT1 fibroblasts compared to Low B4GALT1 fibroblasts were derived through the utilization of the FindMarkers or FindAllMarkers function, employing default parameters and the Wilcoxon rank-sum test, unless explicitly specified. DEGs with an adjusted P-value below 0.05 were selectively retained for subsequent analysis. To curtail the number of genes, the least absolute shrinkage and selection operator (lasso) methodology (coefficient = 0.038) was employed. Subsequently, a Multivariate Cox regression analysis, utilizing the stepwise regression method, was executed to establish a GRGs-based risk signature. Patient stratification into low- and high-risk groups was accomplished through zero-mean normalization. The predictive efficacy of the risk signature was assessed via the timeROC package, facilitating Receiver Operating Characteristic (ROC) analysis. The results conclusively indicated that the risk signature bore significant predictive value concerning patient prognosis.

### 2.9 Immune landscape analysis

The correlation between the prognostic signature and the Tumor Immune Microenvironment (TIME) underwent comprehensive scrutiny through various algorithms, including CIBERSORT, EPIC, MCPCOUNTER, and TIMER. Stromal scores, immune scores, and estimate scores were meticulously computed utilizing the “estimate” R package, facilitating the assessment of variances within the patient’s tumor microenvironment. Additionally, the proportions of 22 distinct immune cell subtypes were estimated through the CIBERSORT algorithm, leveraging data from the GSE27020 cohort. Further investigation delved into the correlation between the genes constituting the signature and the immune score, shedding light on the profound impact exerted by these genes on immune-related functionalities.

### 2.10 Cell lines culture of HNSCC cells and cell transfection

All patients provided explicit informed consent prior to their enrollment in the research initiative. Sample collections adhered to procedures sanctioned by the Ethics Committee of Cancer Hospital and Shenzhen Hospital, Chinese Academy of Medical Sciences (KYLX2021-54). Cell lines pertaining to head and neck squamous cell carcinoma (HNSCC), namely HN-5 cells and UMSCC-47 cells, were procured from the American Type Culture Collection (ATCC). The cultivation of all cells employed RPMI 1640 media (Gibco, USA), supplemented with 10% fetal bovine serum (HyClone Sera, USA) and 1% penicillin‐streptomycin (Sangon Biotech, China). Cellular maintenance occurred within an atmosphere sustained at 37°C with 5% CO2. The SMS siRNA expression vector and the scrambled siRNA nontarget control were acquired from Genewiz (China). Plasmids underwent transfection utilizing Lipofectamine 3000 (Thermo Scientific, USA), following the manufacturer’s prescribed protocols. Furthermore, oligonucleotides used in research were presented in **Supplementary Table S2**.

### 2.11 RNA extraction and quantitative real-time polymerase chain reaction

Total RNA was isolated from the cellular specimens employing TRIzol, following the guidelines stipulated by the manufacturer (15596018, Thermo). Subsequent to this, complementary DNA (cDNA) synthesis transpired through the utilization of the PrimeScript TMRT kit (R232-01, Vazyme). Real-time polymerase chain reaction (RT-PCR) was conducted utilizing the SYBR Green Master Mix (Q111-02, Vazyme). The mRNA expression levels for each target were normalized to the GAPDH mRNA levels, and the quantification of expression levels was executed employing the 2-ΔΔCT method.

### 2.12 Colony formation

We planted transfected head and neck cancer cells in a 6-well plate, with 800 cells per well. After 2 weeks, we observed the number of colonies formed by each group of cells. The cells were fixed, stained, and then counted.

### 2.13 Wound-healing assay and transwell assay

The transfected cells were inoculated in 6-well plates and cultured within a controlled cell incubator until they achieved 95% confluence. Subsequently, each well underwent a delicate scraping process using a sterile 200 ml plastic pipette tip, and any non-adherent cells and debris were rinsed twice with phosphate-buffered saline (PBS). The extent of the scratch wounds was quantified utilizing the Image J program, with photographic documentation captured at both the 0-hour and 48-hour time points. For the experiments pertaining to cell migration, treated HN-5 cells and UMSCC-47 cells (2×104) were incubated in the upper chamber of 24-well plates for a duration of 48 hours. Following the removal of cells from the upper surface, the remaining cells on the lower layer were fixed with 4% paraformaldehyde and stained with 0.1% crystal violet (Solarbio, China).

### 2.14 Statistical analysis

All statistical analyses were executed utilizing the R software (version 4.1.3). The Wilcoxon test was employed for the comparative analysis of two groups, while correlation matrices were scrutinized through the Spearman or Pearson correlation, as deemed appropriate. Survival disparities, as depicted by Kaplan-Meier curves, were evaluated employing the Log-rank test, wherein statistical significance was established at a p-value less than 0.05.

## 3 Results

### 3.1 Enrichment analysis based on transcriptome sequencing

**Figure 1:**
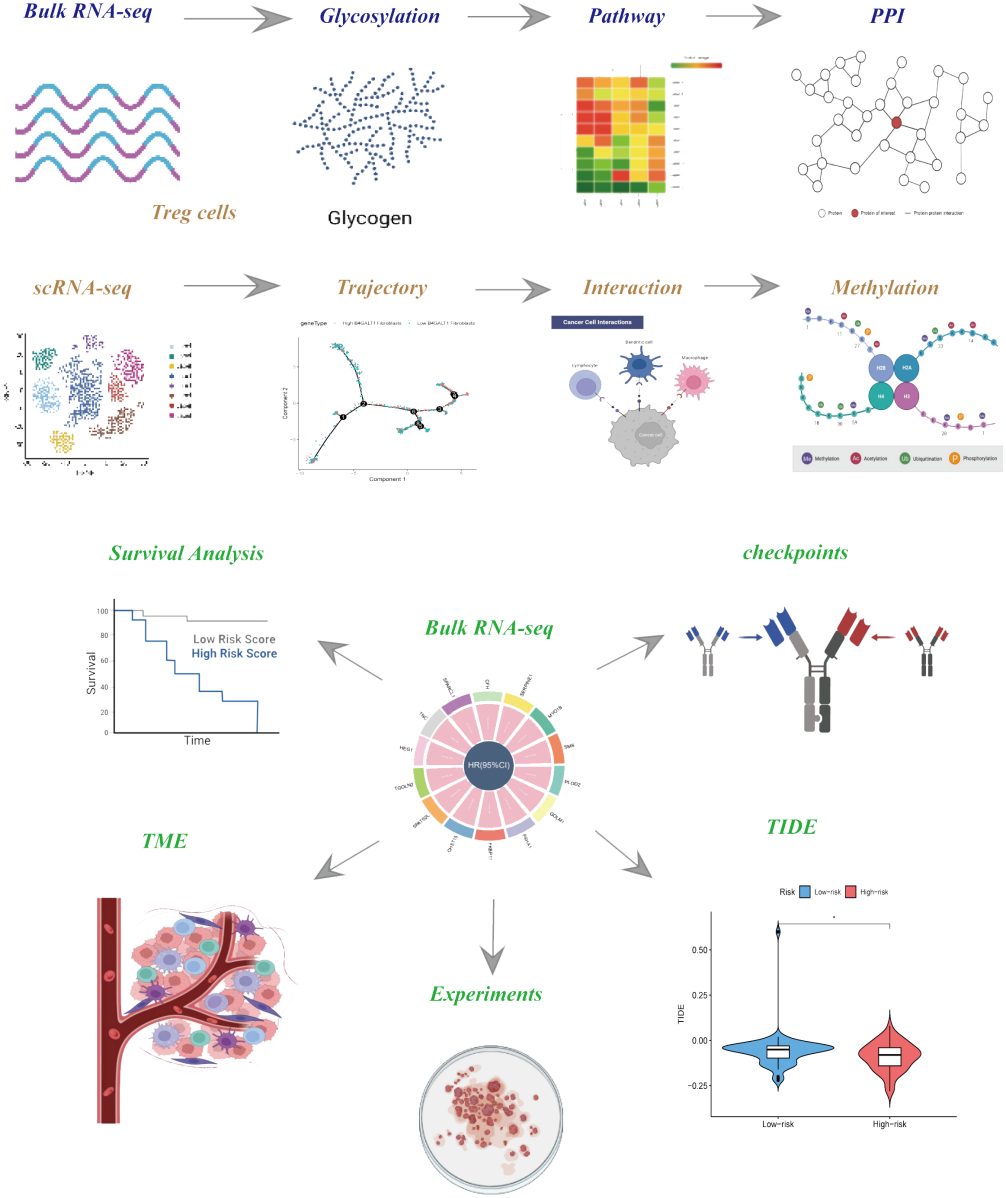
The flow chart of this study.

**Figure 2:**
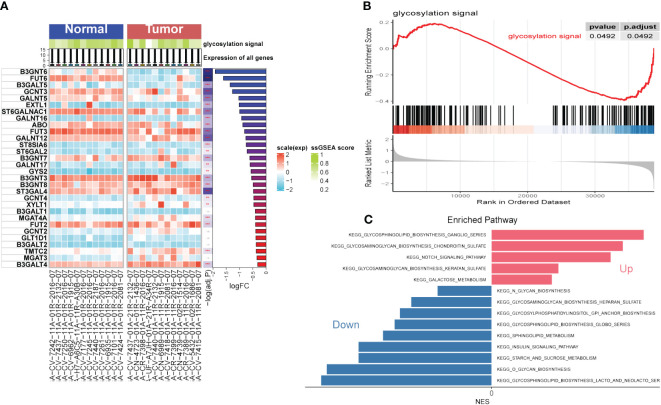
Gene Set Enrichment Analysis (GSEA). **(A)** Single Sample Gene Set Enrichment Analysis (ssGSEA) of Head and Neck Squamous Cell Carcinoma (HNSCC). **(B)** GSEA of HNSCC focused on glycosylation signal. **(C)** Kyoto encyclopedia of genes and genomes (KEGG) of HNSCC. (**P < 0.01; ***P < 0.001)

**Figure 3:**
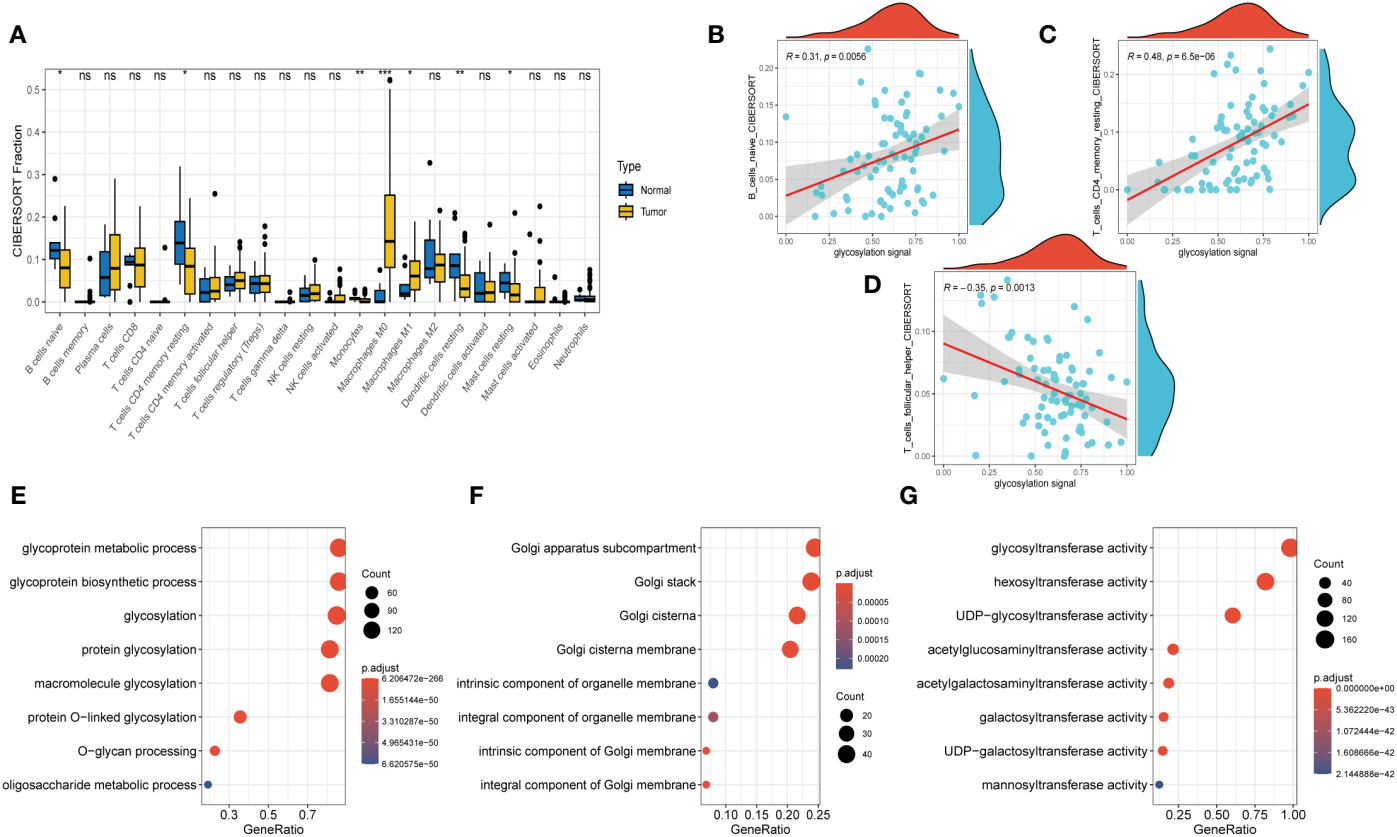
Immune Infiltrations Analysis and GO Analysis. **(A)** Comparison of proportions of 22 immune-related cells between tumor and normal groups. **(B–D)** Correlations between several immune-related cells and glycosylation signal. **(E)** GO-BP analysis **(F)** GO-CC analysis **(G)** GO-MF (E)KEGG analysis. (Wilcox. Test, *P < 0.05; **P < 0.01; ***P < 0.001; ns, not significant).

The flow chart of the study is presented in **Figure 1**. To explore the influence of glycosylation on HNSCC, the data of HNSCC was downloaded and further analyzed from TCGA, which contained 116 malignant samples as well as 12 normal ones. Meanwhile, 184 glycosylation-related genes were obtained from GSEA (Gene Set Enrichment Analysis). As was depicted in the heatmap (**Figure 2A**), the expression of first 30 glycosylation-related genes was exhibited with 12 paired tumor-and-normal samples. Then, GSEA analysis was conducted, which indicated that glycosylation signal was significantly enriched during these samples (**Figure 2B**). Furthermore, these RGRs were enrolled into differential analysis (results of differential analysis were presented in **Supplementary Table S1**) and **Figure 2C** demonstrated that up-regulated genes were overwhelmingly enriched in pathways including glycosphingolipid biosynthesis ganglio series, notch signaling pathway and so on. On the other hand, down-regulated genes were mostly enriched in N-glycan biosynthesis, glycosphingolipid biosynthesis heparan sulfate, etc. Both the notch signaling pathway and were identified to contribute to the initiative, proliferation and migration of several cancers (22, 23). Likewise, results of GO analysis also verified that these GRGs were obviously enriched in glycosylation related pathways (**Figures 3E–G**).

### 3.2 Immune infiltrations exploration in transcriptome sequencing

After conducting the enrichment analysis, the transcriptome data was further enrolled for immune infiltrations exploration. As depicted in **Figure 3A**, slight difference of immune infiltrations was identified between tumor specimens and normal ones. Higher proportions of immune-related cells were found enriched in normal samples compared with those malignant ones, such as naïve B cells, CD4^+^ T cells (memory resting), monocytes, resting dendritic cells, and resting mast cells. Conversely, more percentage of macrophages (M0, M1) were observed in the normal samples. Moreover, method of ‘Spearman’ was utilized to explore the relationship between immune infiltrations and glycosylation. The results manifested that proportions of naïve B cells and CD4^+^ T cells (memory resting) were positively related to glycosylation, while T cells (follicular helper) were negatively correlated with glycosylation (**Figures 3B–D**). Results of disparities of immune cells proportions between different samples were presented in **Supplementary Figure S1**.

### 3.3 PPI (Protein-protein interaction)

The fundamental essence of nearly every biological process is encapsulated within proteins and their intricate interactions. To obtain the core genes that play a pivotal role in the glycosylation of HNSCC, PPI network was performed. Well exhibited in **Supplementary Figure S2A**, these GRGs have extensive links between each other and B4GALT1 was found to have the most edges in the network, according to which, we infer that B4GALT1 might play an indispensable role in the HNSCC’s glycosylation. Besides, relevant parameters used in the PPI network were displayed in **Supplementary Figure S2B**.

### 3.4 RGRs expression in scRNA samples

**Figure 4:**
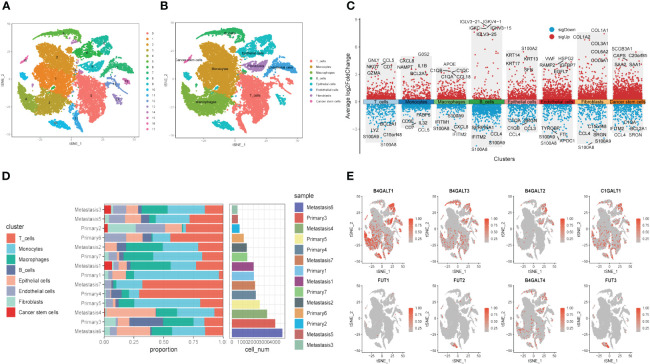
Glycosylation-related genes (GRGs) expression in scRNA data of HNSCC patients. **(A)** tSNE plots of distribution of 18 clusters. **(B)** tSNE plots of distribution of 8 clusters after annotation. **(C)** Volcano plot of the top5 marker gene expression of subgroups. **(D)** Histogram showing cell numbers in main clusters and samples. **(E)** tSNE plots exhibiting eight GRGs expression.

Next, to explore the expression pattern of GRGs in different cells, scRNA sequencing of HNSCC (GSE234933) was obtained from GEO database, from which 7 primary tumor samples as well as 7 metastatic ones were selected for subsequent analysis. Totally, 28736 cells were acquired from the scRNA-seq data after initial screening. Results of data preprocessing details were presented in **Supplementary Figure S3**. To begin with, 18 subpopulations were identified after conducting log-normalization and dimensionality reduction (**Figure 4A**). Then, we performed the cell annotation and obtained 8 types of cells, including T cells, B cells, Monocytes, Macrophages, Endothelial cells, Epithelial cells, Cancer stem cells, and Fibroblasts (**Figure 4B**). Furthermore, upon conducting the method of ‘FindVariableFeature’, 9996 DEGs (different expression genes) were acquired based on the 8 clusters. The volcano plot depicted the top5 DEGs (**Figure 4C**). According to these DEGs, GSEA analysis was performed, the results demonstrated that DCN, LUM, COL1A1 and CXCL14 were significantly enriched in Fibroblasts and remarkably correlated with several pathways, such as collagen fibril organization, extracellular matrix organization and so on (**Supplementary Figure S4**). Besides, the histograms exhibited the cell proportions of the 8 clusters in each sample (**Figure 4D**), which demonstrated that the sample metastasis5 acquired the highest cell proportions and sample primary7 shared the most T cells. The last, 8 GRGs that exert crucial impacts in the former PPI network were presented in tsne plots (**Figure 4E**). The expression of B4GALT1 was found significantly high in Fibroblasts, Macrophages and Epithelial cells.

### 3.5 Glycosylation’s influence in different cells

**Figure 5:**
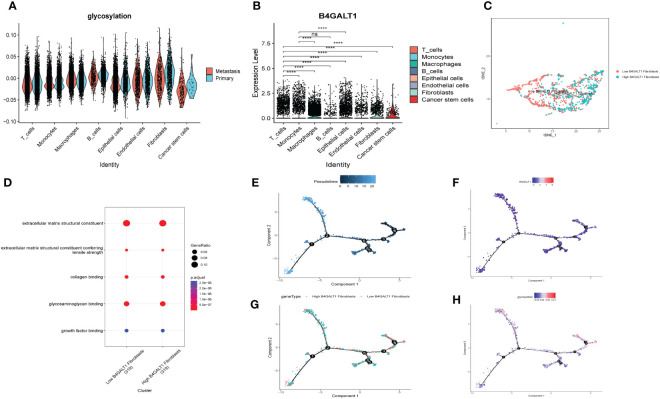
Glycosylation of HNSCC based on fibroblasts. **(A)** Disparities of glycosylation between metastasis and primary HNSCC samples in different cells. **(B)** Expression level of B4GALT1 in different cells. **(C)** tSNE plots of distributions of ‘Low B4GALT1 Fibroblasts’ and ‘High B4GALT1 Fibroblasts’ after clustering. **(D)** GO-BP analysis of ‘Low B4GALT1 Fibroblasts’ and ‘High B4GALT1 Fibroblasts’ clusters. Trajectory of Pseudotime **(E)**, expression level of B4GALT1 **(F)**, ‘Low B4GALT1 Fibroblasts’ and ‘High B4GALT1 Fibroblasts’ clusters **(G)**, glycosylation level **(H)**. (Wilcox. Test, ****P < 0.0001; ns, not significant).

Similar to former findings, expression of B4GALT1 was identified dramatically high in Fibroblasts, Macrophages and Epithelial cells (**Figure 5B**). With the method of ‘AddModuleScore’, the violin plots exhibited that there was little difference of glycosylation between primary tumor samples and the metastasis ones (**Figure 5A**). Considering the high expression of B4GALT1 in Fibroblasts, the cluster of Fibroblasts was screened out and further classified into ‘low B4GALT1 Fibroblasts’ and ‘High B4GALT1 Fibroblasts’ subgroups (**Figure 5C**). Then, results of GO enrichment analysis revealed that both the two subgroups were significantly correlated with extracellular matrix structural constituent (**Figure 5D**). Next, we performed monocle2 to probe into the fibroblast’s potential developmental trajectory. Interestingly, we found that high expression of B4GLTA1 was obviously located at the ending point of the developmental trajectory (**Figures 5E, F**). Similarly, ‘High B4GLAT1 Fibroblasts’ was identified at the end of developmental trajectory (**Figure 5G**). Thus, we infer that B4GALT1 might impede the progression of HNSCC. By converse, **Figure 5H** displayed that low proportion of glycosylation was relatively enriched at the beginning point of developmental trajectory, demonstrating that glycosylation may contribute to the initiation and progression of HNSCC.

### 3.6 Specific cellular interactions between fibroblasts and the others

**Figure 6:**
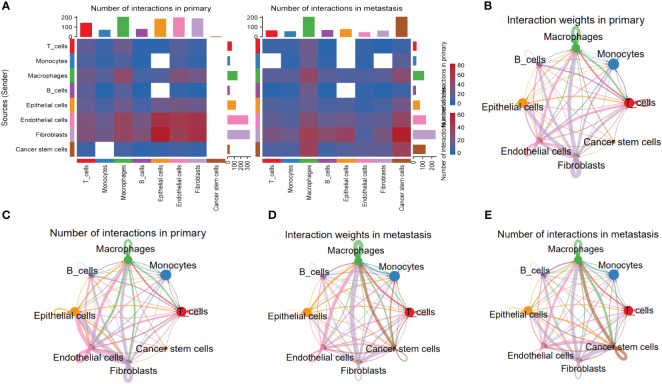
Specific cellular interactions between fibroblasts and the others. **(A)** Heatmap displaying number of interactions in primary and metastasis HNSCC samples. **(B)** Cycle graph showing interaction weighs in primary HNSCC samples. **(C)** Cycle graph showing number of interactions in primary HNSCC samples. **(D)** Cycle graph showing interaction weighs in metastasis HNSCC samples. **(E)** Cycle graph showing number of interactions in metastasis HNSCC samples.

To explore the heterogeneity of cell-cell interactions between primary tumor samples and the metastatic ones, series of ligand-receptor (L-R) pairs obtained from CellphoneDB database were utilized to predict potential interactions among eight major cell types. Compared with metastasis tumor samples, the primary ones had more sufficient interaction pairs, especially in Fibroblasts, Macrophages, and Endothelial cells (**Figure 6A**). Besides, these cycle graphs (**Figures 6B–E**) demonstrated that in spite of less interaction numbers, metastatic samples had more intensive interaction weights, indicating that cell-cell interactions were vividly involved during the progression of malignant cells.

### 3.7 DNA methylation analysis of B4GALT1 in HNSCC

**Figure 7:**
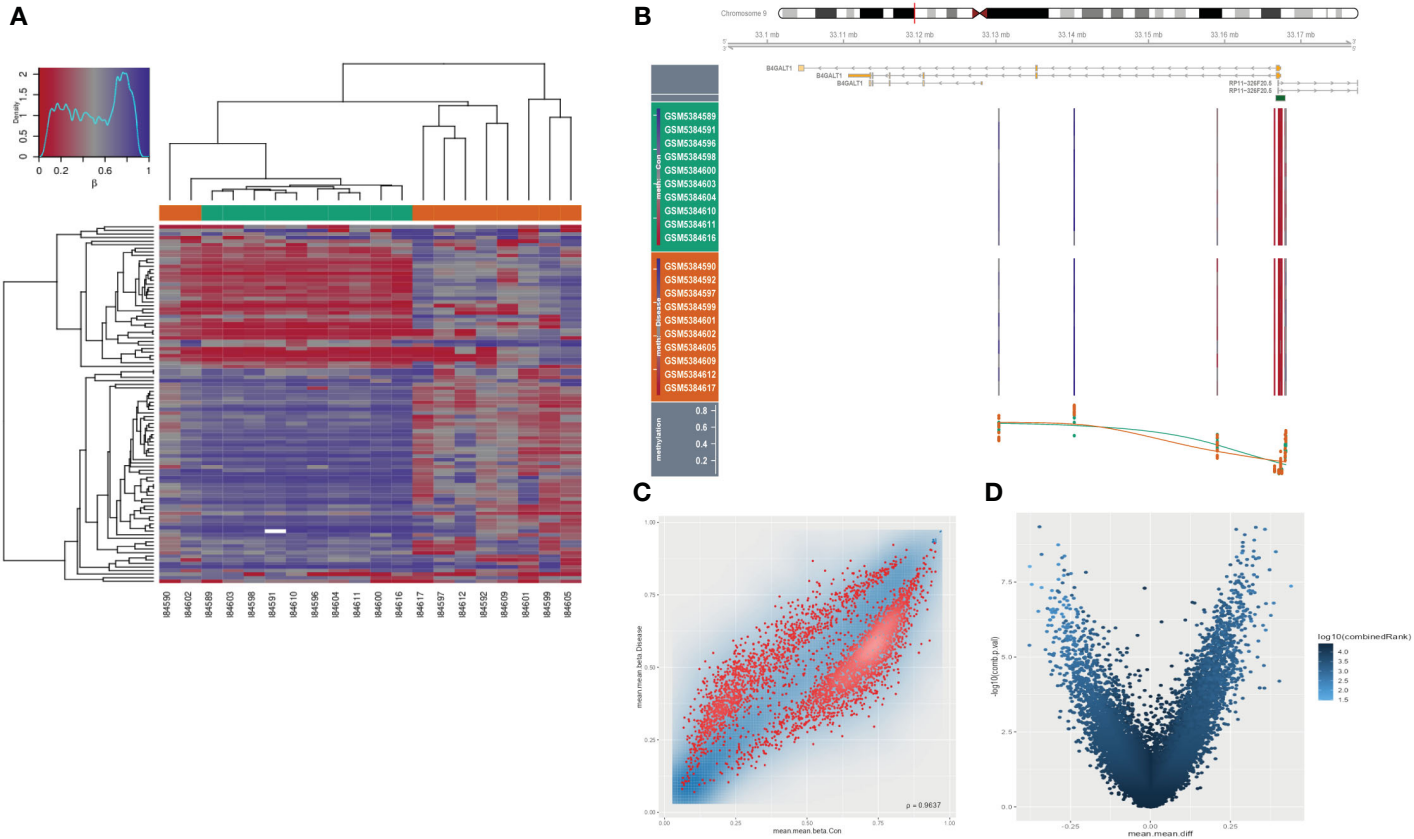
Methylation analysis in HNSCC. **(A)** Heatmap presenting disparities of methylation between tumor and normal samples. **(B)** Methylation of B4GALT1 in HNSCC. **(C, D)** Volcano plots showing differences of methylation level between different genes.

As an extremely malignant tumor, HSCC has been identified significantly correlated with epigenetics. Thus, we further explored the B4GALT1 methylation in HSCC with GEO database (GSE178218). The heatmap presented the differences in methylation level between tumor samples and the normal ones (**Figure 7A**), revealing that normal samples might have a relatively high methylation level. Besides, we could infer that methylation level of B4GALT1 in normal specimens was slightly higher than that in malignant samples based on **Figure 7B**. Moreover, results of disparity of methylation level between different genes were exhibited in **Figures 7C, D**.

### 3.8 Risk signature construction and validation

**Figure 8:**
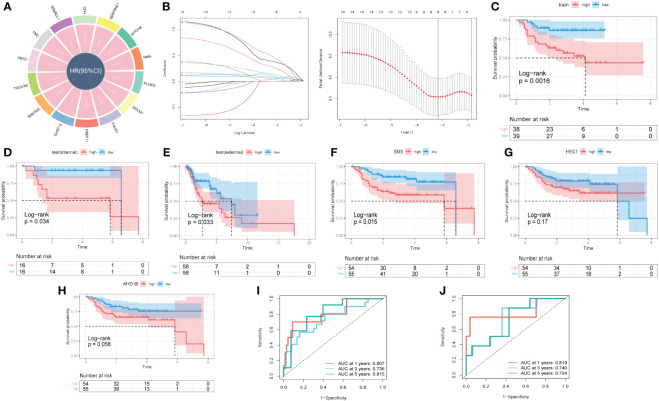
A novel risk signature developed based on several GRGs. **(A)** Results of univariate cox regression based on these GRGs. **(B)** Each independent variable’s trajectory and distributions for the lambda. **(C)** K-M curves of the risk signature in training cohort. **(D)** K-M curves of the risk signature in internal test cohort. **(E)** K-M curves of the risk signature in external test cohort. **(F)** K-M curves of the gene SMS. **(G)** K-M curves of the gene HEG1. **(H)** K-M curves of the gene MYO1B. **(I)** ROC curves of the risk signature in training cohort. **(J)** ROC curves of the risk signature in test cohort.

With the scRNA-seq data, DEGs between ‘low B4GALT1 Fibroblasts’ and ‘High B4GALT1 Fibroblasts’ cells were acquired. The prognosis of these genes was further evaluated using univariate cox regression. As presented in **Figure 8A**, 14 RGRs were identified significantly correlated with the prognosis of HNSCC. To diminish the number of genes, we executed Lasso Cox regression analysis, yielding a set of three genes. Ultimately, employing the stepwise regression methodology, we developed the risk signature subsequent to a comprehensive multivariate Cox regression analysis (**Figure 8B**). The signature comprised of 3 genes, namely spermine synthase (SMS), heart development protein with EGF like domains 1 (HEG1) and myosin IB (MYO1B). Utilizing z-score normalization, we computed the risk scores for each patient, stratifying individuals into categories of high-and-low risk. The data of GSE27020 was used as training cohort while the testing cohort was constructed based on TCGA database. Kaplan-Meier survival analysis elucidated those characterized as low-risk exhibited markedly superior survival outcomes in contrast to their high-risk counterparts, which could be validated not solely within the training cohort but also extensible to both internal and external cohorts (**Figures 8C–E**). Moreover, based on the TCGA and GEO cohorts, the Area Under the Curve (AUC) metrics of the signature for survival spanning 1, 3, and 5 years were deemed gratifying, thereby signifying the model’s exemplary prognostic efficacy (**Figures 8I, J**). Besides, these 3 genes were enrolled into Kaplan-Meier survival analysis and the results indicated that both of them were risk genes, however, only gene SMS was identified with p-value < 0.05 (**Figures 8F**–**H**). We also presented the distribution of risk score, patient survival status, and expression of hub genes in the training cohort in **Supplementary Figure S5A**. Similarly, the results of both internal and external cohorts were shown in **Supplementary Figures S5B, C**, illustrating that the risk signature can excellently predict the prognosis of HNSCC patients.

### 3.9 Landscape of immune infiltrations and correlations between signature and immunity

**Figure 9:**
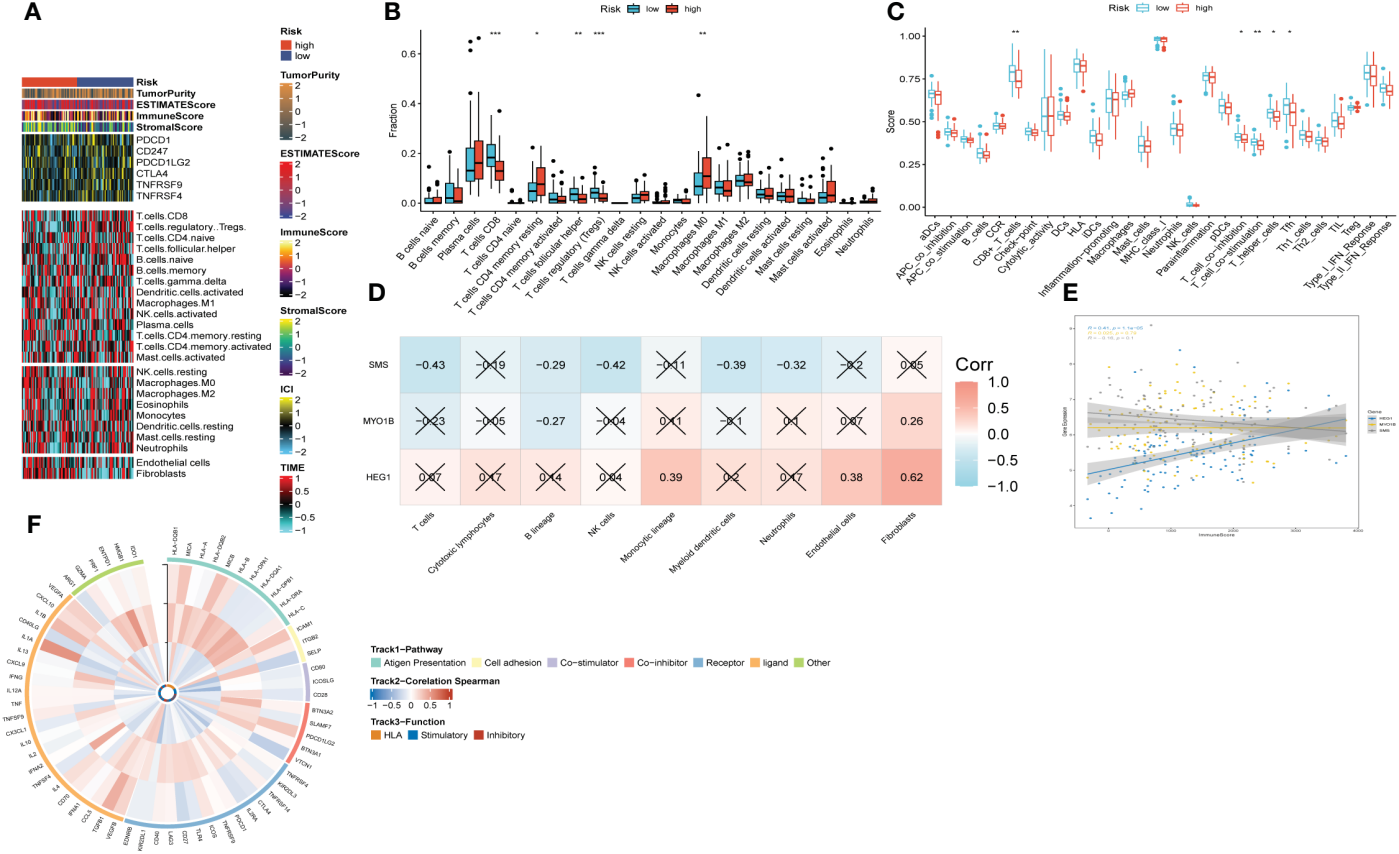
The immune infiltrations analysis based on risk signature. **(A)** Heatmap of results on immune cells of tumor microenvironment (TME) in HNSCC with multialgorithm. **(B)** Comparison of proportions of 22 immune-related cells between high-and-low-risk groups. **(C)** Comparison of proportions of immune-related functions between high-and-low-risk groups. **(D)** Correlations between nine hub genes and 22 immune-related cells. **(E)** Correlations between the three hub genes and immune score. **(F)** The correlation analysis between three hub genes and 75 immune-associated genes.

Upon scrutinizing the landscape of immune and stromal cell infiltrations within both cohorts characterized by low and high risk, our investigation delineates the discernible disparity, showcasing individuals within the high-risk stratum manifest elevated proportions of immune and stromal cell infiltrations in comparison to their low-risk counterparts (**Figure 9A**). Employing the CIBERSORT algorithm for a comprehensive analysis, we computed the immune cell proportions distinguishing the high-risk and low-risk groups (as illustrated in **Figures 9B, C**). Our findings underscore a noteworthy observation: individuals within the high-risk category exhibit a statistically significant augmentation in the proportions of CD4 T cells (memory resting) and Macrophages (M0). Conversely, within the low-risk cohort, an enrichment is observed in CD8 T cells, T cells (follicular helper), and Tregs. We subsequently probed into the intricate interplay between risk-associated genes and the immune milieu (**Figures 9D, E**). Our findings evinced a notably affirmative correlation between HEG1 and kinds of immune cells. Conversely, both SMS and MYO1B exhibited a discernibly inverse association with immune cells. Ultimately, **Figure 9F** elucidated the interrelationship among the triad of risk genes and the seventy-five genes germane to the immune system.

### 3.10 Immunotherapy response prediction

**Figure 10:**
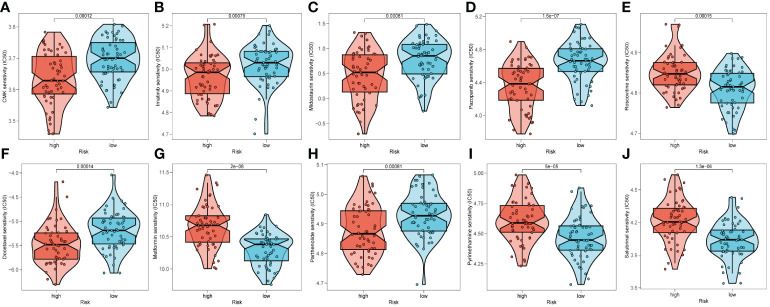
Prediction of chemotherapy drug sensitivity in ESCC patients based on different groups, including CMK **(A)**, Imatinib **(B)**, Midostaurin **(C)**, Pazopanib **(D)**, Roscovitine **(E)**, Docetaxel **(F)**, Metformin **(G)**, Parthenolide **(H)**, Pyrimethamine **(I)**, and Salubrinal **(J)**.

To predict the response to immunotherapy, we developed TIDE based on the risk signature. The results reflected patients in high-risk group had higher score of Exclusion (**Supplementary Figure S6C**) while shared lower MSI scores (**Supplementary Figure S6D**) than those in low-risk group. However, there was little difference found in terms of Dysfunction (**Supplementary Figure S6B**). In summary, patients in low-risk group had higher TIDE scores (**Supplementary Figure S6A**), indicating that those patients probably response poorly to immunotherapy based on ICIs. After comparing the efficacy of diverse chemotherapeutic agents across distinct cohorts, we discerned individuals affiliated with the high-risk category manifested augmented IC50 values for chemotherapeutic substances such as Roscovitine, Metformin, Pyrimethamine, and Salubrinal (**Figures 10E, G, I, J**). Conversely, patients within the low-risk category were noted to exhibit heightened responsiveness to CMK, Imatinib, Docetaxel, and the like (**Figures 10A–D, F, H**).

### 3.11 *In vitro* biological function in HNSCC cells

**Figure 11:**
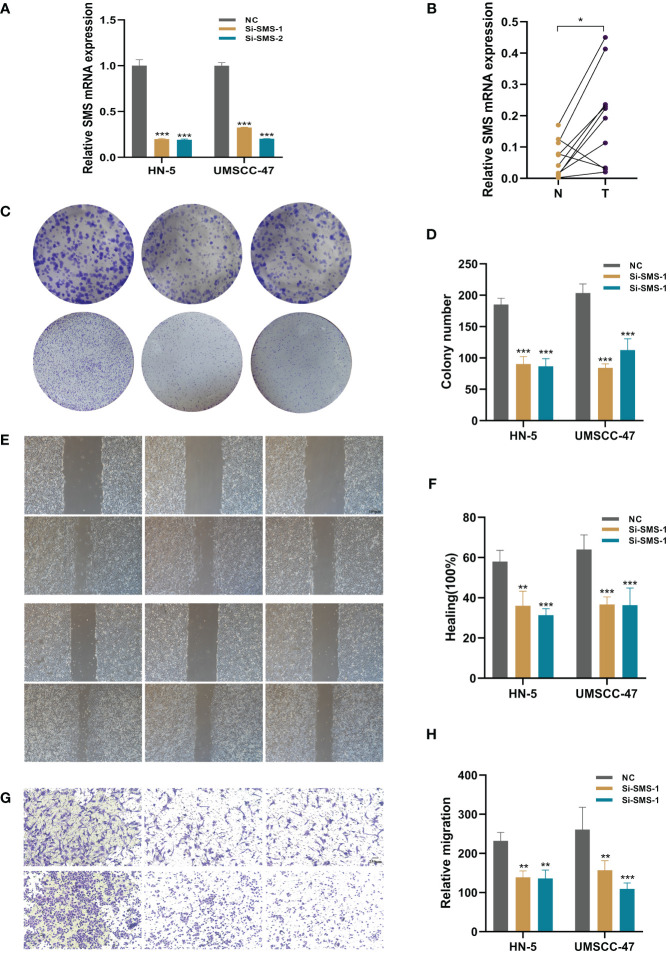
The role of SMS in HNSCC. **(A)** SMS expression in NC group, HN-5 cells and UMSCC-47 cells following treatment with siRNA sequences (Si-1 and Si-2) **(B)** The expression of SMS in HNSCC tissue and normal tissue of patients. t-test was used to compare the expression of genes between normal and tumor. **(C, D)** The results of the plate cloning assay of SMS expression in NC group, HN-5 cells and UMSCC-47 cells following treatment with siRNA sequences **(E, F)** Scratch-wound healing assay depicted that a significantly slower wound healing rate was observed in cells with a decreased expression of SMS. **(G, H)** Transwell assay showed that downregulation of SMS expression inhibited the migration and invasion capacity of HNSCC cells. To demonstrate the accuracy and reproducibility of the results, all experiments were repeated in two HNSCC (HN-5, UMSCC-47) cell lines and all data were presented as the means ± SD of three independent experiments, *P < 0.05, **P < 0.01, ***P < 0.001.

In order to further elucidate the role of SMS in HNSCC, we conducted *in vitro* investigations to scrutinize the functionality of SMS in HNSCC cells. We quantified the level of SMS expression after 24 hours of transfection using quantitative Reverse Transcription Polymerase Chain Reaction (qRT-PCR) to evaluate the effectiveness of siRNA-mediated SMS knockdown in HN-5 cells and UMSCC-47 cells. In comparison to the NC group, we observed a significant reduction in SMS expression in HN-5 cells and UMSCC-47 cells following treatment with siRNA sequences (Si-1 and Si-2) (P < 0.001) (**Figure 11A**). Correspondingly, in the validation of tissues from 10 patients, the results showed that the expression level of SMS in tumor tissue was higher compared to adjacent non-cancerous tissue. (**Figure 11B**). The results of the plate cloning assay provided additional evidence that the inhibition of SMS expression hindered the proliferation of HN-5 cells and UMSCC-47 cells relative to the NC group (**Figures 11C, D**). This suggests that SMS may play an indispensable role in the proliferation of HNSCC cells. The scratch-wound healing experiment also yielded congruent results; wherein decreased SMS expression led to a noteworthy deceleration in the rate of wound healing in cells (**Figures 11E, F**). The transwell experiments confirmed that SMS knockdown considerably reduced the migration and invasion of HN-5 cells and UMSCC-47 cells (**Figures 11G, H**). To ensure the accuracy and consistency of the results, all tests were conducted in two HNSCC cell lines (HN-5 and UMSCC-47), and all data were presented as means with standard deviations from three independent experiments. *P < 0.05, **P < 0.01, ***P < 0.001.

## 4 Discussion

It is widely acknowledged that head and neck squamous cell carcinoma (HNSCC) is characterized by an exceedingly high degree of malignancy, posing a significant threat to human health (1, 24). Despite the implementation of comprehensive interventions encompassing surgery, radiotherapy, chemotherapy, and the like, the prognosis for HNSCC remains markedly unsatisfactory (25). In addition to genetic and epigenetic alterations, aberrant glycosylation in cancer is increasingly being acknowledged as a distinctive hallmark. Accumulating evidence suggests that hyperactive glycan synthesis, uptake, and storage pathways contribute to tumorigenesis. Therefore, the inhibition of glycosylation pathways presents itself as a potential therapeutic target in the context of cancers (26, 27). Nevertheless, the connection between glycosylation and the pathological progression of cancer is not sufficiently elucidated.

Firstly, KEGG analysis was performed based on the glycosylation-related genes in transcriptome data. Interestingly, notch signal pathway was identified significantly correlated with up-regulated genes, which has been verified to play a pivotal role in initiative and progression of HNSCC (28). Down-regulated genes were found obviously enriched in N glycan biosynthesis and it has been reported that several types of N-glycome could be observed in HNSCC cell lines (29), revealing that N glycan biosynthesis might have impacts on biological behavior of HNSCC. We further developed the immune infiltrations analysis, which demonstrated that glycosylation was positively associated with B cells (naive) and CD4^+^ T cells (memory resting) and negatively related to T cells (follicular helper), that play crucial roles in tumor immune microenvironment.

Considering that most of proteins act in multimolecular complexes instead of isolation within cells, PPI network was conducted. Our results showed that B4GALT1 had the most edges and nodes, striking on us that B4GALT1 might play an indispensable role in the network. The proteins identified in the PPI network, particularly those directly interacting with B4GALT1, likely exert complementary or synergistic effects on HNSCC biology. For example, proteins involved in the regulation of cell cycle progression, apoptosis, DNA repair, and epithelial-mesenchymal transition (EMT) may cooperate with B4GALT1 to promote tumor growth, invasion, and metastasis. Additionally, proteins implicated in drug metabolism, resistance, and detoxification pathways may contribute to therapeutic resistance in HNSCC patients. Grasping the intricacies of these PPIs and their dynamic nature constitutes one of the paramount challenges in the realms of cell and cancer cell biology, offering the potential for the development of innovative therapeutics (30).

Single-cell RNA sequencing (scRNA-seq) emerges as a formidable methodology for deconstructing the intricacies inherent in solid tumors, facilitating the discernment of cellular diversity and the delineation of heterogeneous phenotypic states with unparalleled granularity (31). HNSCC is characterized by substantial intra-tumoral heterogeneity, encompassing diverse cell populations with distinct molecular profiles and functional roles. By delineating the glycosylation patterns across different cell types, including tumor cells, immune cells, fibroblasts, and endothelial cells, our scRNA-seq analysis offers unprecedented insights into the heterogeneity of glycosylation landscapes within the tumor microenvironment. This heterogeneity likely contributes to the diverse phenotypic behaviors observed in HNSCC, such as tumor growth, invasion, metastasis, and response to therapy (32). Containing malignant cells and a bunch of non-malignant cells, cancers stand for complex ecosystems, embedded in variant extracellular matrix. The tumor microenvironment (TME) comprises of several immune cell types, including endothelial cells, cancer-associated fibroblasts, pericytes, and diverse tissue-resident cell types (33). Single-cell RNA sequencing has been widely applied to probe into the intricates of TME, shedding new light on the management of cancers (34). For example, high levels of cytotoxic T lymphocytes (CTLs) within the tumor microenvironment have been associated with improved survival in HNSCC patients. CTLs play a crucial role in recognizing and eliminating cancer cells, thus inhibiting tumor growth and metastasis (35). Conversely, regulatory T cells (Tregs) may suppress anti-tumor immune responses, promoting tumor immune evasion and progression (36). Lately, single cell analyses have unveiled the intricacies of tumor-infiltrating myeloid cells, encompassing tumor-associated macrophages (TAMs) and dendritic cells (DCs), across various malignancies (37, 38). (TAMs) represent a significant component of the immune infiltrate in HNSCC. Depending on their polarization state, TAMs can exhibit either pro-tumorigenic (M2-like) or anti-tumorigenic (M1-like) properties. M2-like TAMs are associated with tumor-promoting activities, including angiogenesis, immunosuppression, and tissue remodeling, whereas M1-like TAMs may exert anti-tumor effects by promoting inflammation and cytotoxicity (39). The biological function of fibroblasts, however, have not been well elucidated so far. Thus, we performed scRNA-seq analyses based on public database to unveil glycosylation’s impact on various cells. It was found that fibroblasts beard the highest level of glycosylation and B4GALT1 was notably enriched in fibroblasts. The following trajectory analyses using ‘Monocle2’ exhibited that the level of glycosylation got higher while the expression of B4GALT1 became lower, disclosing that glycosylation might incur migration and invasion of HNSCC. Moreover, the specific cellular interactions showed that fibroblasts had intensive interactions with other cells, especially Epithelial cells or Cancer stem cells, illustrating that Fibroblasts exert great influence on TME of HNSCC. To notice, it has been discovered that cancer-associated fibroblasts (CAFs) located in primary and metastatic neoplasms exhibit remarkable versatility, plasticity, and resilience, actively participating in the intricate dynamics of cancer progression through intricate interplays with diverse cell types within the tumor microenvironment (40).

Apart from glycosylation, epigenetic could also regulate the tumor invasion and metastasis. Epigenetic features commonly deviate from the norm in neoplastic cells. Human malignancies frequently manifest distinctive alterations in DNA methylation, encompassing global hypomethylation and locus-specific hypermethylation (41). Histomorphology has persistently stood as a cornerstone in cancer diagnosis within anatomic pathology for numerous years. DNA methylation profiling emerges as an evolving adjunctive instrument poised to augment the precision of pathological diagnoses (42). Methylation alterations in the promoter region of B4GALT1 may lead to aberrant gene silencing or overexpression, thereby influencing glycosylation patterns and tumor behavior in HNSCC. For instance, hypermethylation of CpG islands within the B4GALT1 promoter may contribute to transcriptional repression and reduced B4GALT1 expression, leading to altered glycosylation profiles associated with tumor progression and therapeutic resistance. In our study, we developed methylation analyses in HNSCC and the results illustrated that the malignant samples tended to have higher level of methylation compared with the normal ones. Besides, slight disparity was discovered in methylation level of B4GALT1 between tumor species and the normal ones. One research argued that both the attenuated expression and promoter hypermethylation of B4GALT1 exert an adverse prognostic influence on colorectal malignancy (43). In summary, we hypothesize that low expression and high level of methylation of B4GALT1 could promote migration and invasion of HNSCC. Despite the extraordinary progresses have been achieved recently in the therapy of HNSCC, there still exists the dilemma: unfavorable curative effects and poor prognosis (44). Lack of early diagnosis and precise treatment give rise to the detrimental outcome. Hence, we developed a risk signature to predict the prognosis and immunotherapy response of HNSCC. The novel signature was composed of three genes, including SMS, HEG1, and MYO1B. Multiple researches have unveiled that SMS is remarkably related with initiative, invasion of several kinds of cancers and can function as a biomarker for prognosis (45–47). Yet, the relationship between SMS and HNSCC has not been well elucidated. In our study, it was found that HNSCC patients with high level expression of SMS encountered significantly unfavorable outcome through the initial bioinformatic analyses. The three genes may function synergistically to promote HNSCC progression by influencing common signaling pathways involved in proliferation, invasion, and immune evasion. For example, dysregulation of SMS may alter lipid metabolism, leading to changes in membrane composition that affect the localization and activity of MYO1B, thereby enhancing cancer cell motility and invasion. Additionally, HEG1-mediated alterations in cell-cell adhesion and signaling may potentiate the effects of MYO1B and SMS dysregulation, further driving HNSCC aggressiveness. Furthermore, the vitro experiments validated that SMS could strikingly promote proliferation, invasion, and migration in HNSCC cell lines. Under the circumstances, SMS was deemed to be obviously correlated with HNSCC prognosis and excellently function as a biomarker. Far from mere conglomerations of malignantly proliferating cells, tumors represent intricately organized complex ecosystems (48). Comprising discrete populations of immune cells within tumor islands, the Tumor Immune Microenvironment (TIME) exhibits a pronounced correlation with the anti-tumor immunological milieu of the Tumor Microenvironment (TME) (49). The TIME has long been recognized as significantly linked to the progression, recurrence, and metastasis of tumors (50). Besides, it has been well established that the features in TIME have tremendous impacts on immunotherapy response in several tumors (51–53). In order to gain deeper insights into the ramifications of our risk signature, we systematically evaluated the immune infiltration status utilizing diverse algorithms. The results revealed that high-risk group had relatively high level of immune infiltrations, implying that this group tend to set up a ‘hot’ tumor state to stimulate the immune system to impute tumor progression. Additionally, analysis of immune infiltration status alongside the risk signature can provide insights into the composition and functional orientation of tumor-infiltrating immune cells. T cells regulatory (Tregs) were observed dramatically enriched in low-risk group, which has been identified correlated with immunotherapy resistance in cancers (54). Results of TIDE analysis reflected patients in low-risk group were more likely prone to immune escae or immunosuppression during anti-tumor immunotherapy. Accordingly, we assume that patients in high-risk group probably response better to anti-tumor immunotherapy.

## 5 Conclusion

Our risk signature based on GRGs autonomously forecasts the prognosis of patients with HNSCC and anticipates their responsiveness to immunotherapeutic interventions. Nevertheless, certain constraints in our investigation necessitate attention. Primarily, the risk signature was formulated utilizing retrospective data derived from publicly available databases. Consequently, a more extensive collection of prospective and multi-center HNSCC cohorts is imperative to mitigate inherent biases. Secondly, our predictive scope was confined to immunotherapeutic responsiveness via the employment of our risk signature. Subsequent investigations are indispensable to assess the potential of our risk signature in predicting responses to diverse precision therapies in forthcoming studies.

## Data availability statement

The datasets presented in this study can be found in online repositories. The names of the repository/repositories and accession number(s) can be found in the article/**Supplementary Material**. The raw data for this study is also accessible at the following link: https://www.jianguoyun.com/p/DYwjjoIQjdemCxj9xa0FIAA.

## Ethics statement

Ethical approval was not required for the studies on humans and animals in accordance with the local legislation and institutional requirements because only commercially available established cell lines were used.

## Author contributions

HM: Writing – original draft, Writing – review & editing. LX: Writing – original draft, Writing – review & editing. BZ: Writing – original draft, Writing – review & editing. ZH: Conceptualization, Data curation, Formal analysis, Funding acquisition, Investigation, Methodology, Project administration, Resources, Software, Supervision, Validation, Visualization, Writing – review & editing. MW: Conceptualization, Data curation, Formal analysis, Funding acquisition, Investigation, Methodology, Project administration, Resources, Software, Supervision, Validation, Visualization, Writing – review & editing. QR: Writing – original draft, Writing – review & editing, Conceptualization, Data curation, Formal analysis, Funding acquisition, Investigation, Methodology, Project administration, Resources, Software, Supervision, Validation, Visualization. SY: Conceptualization, Data curation, Formal analysis, Funding acquisition, Investigation, Methodology, Project administration, Resources, Software, Supervision, Validation, Visualization, Writing – original draft, Writing – review & editing. HS: Writing – original draft, Writing – review & editing.
