# Supplementary figures and images for: Comprehensive investigation into the influence of glycosylation on head and neck squamous cell carcinoma and development of a prognostic model for risk assessment and anticipating immunotherapy
Source: Front Immunol. 2024 Mar 18;15:1364082. doi: 10.3389/fimmu.2024.1364082 (PMC10982401; doi:10.3389/fimmu.2024.1364082)

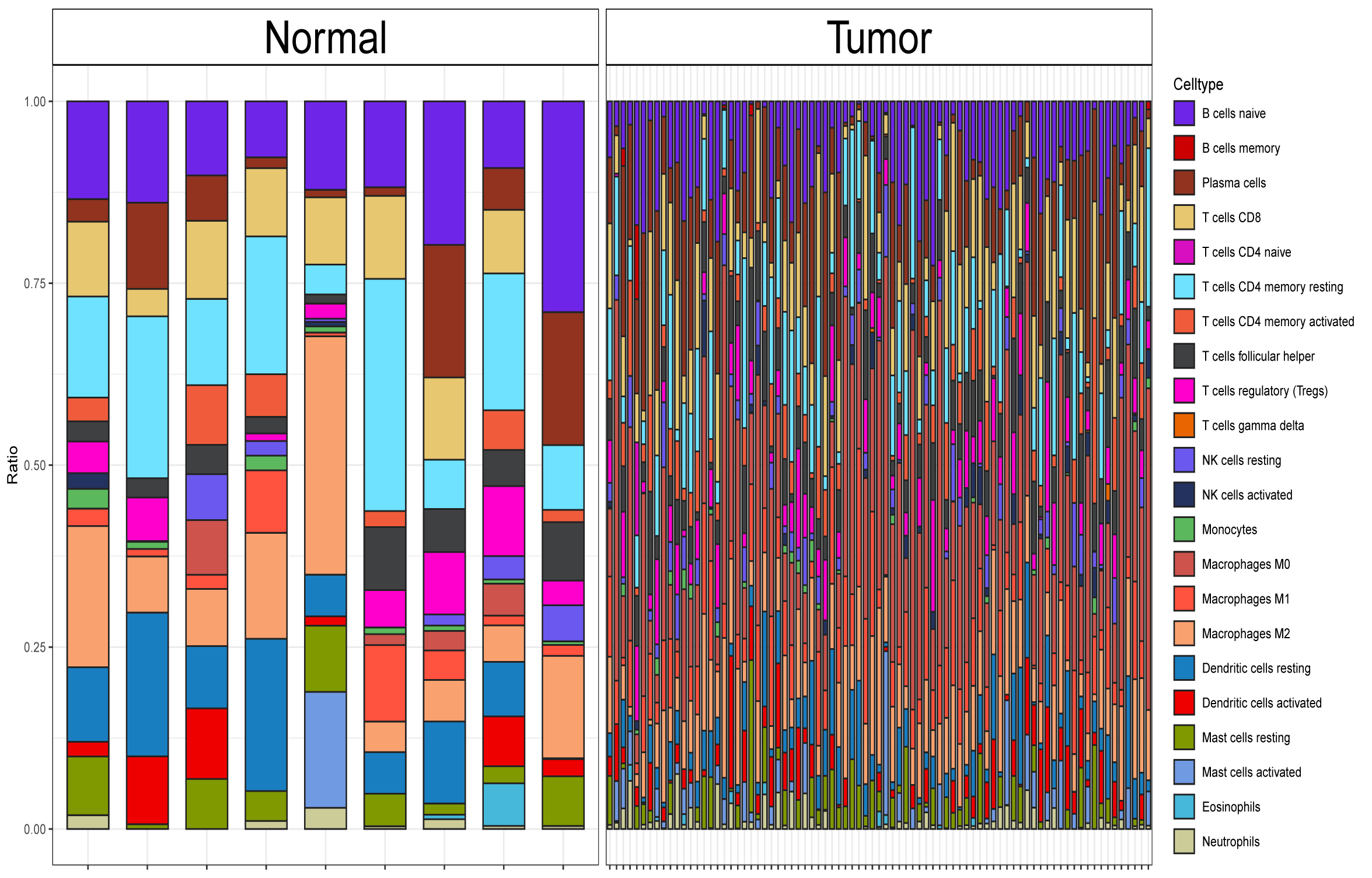

Supplement: Supplementary file 2 [file Image_1.tif]

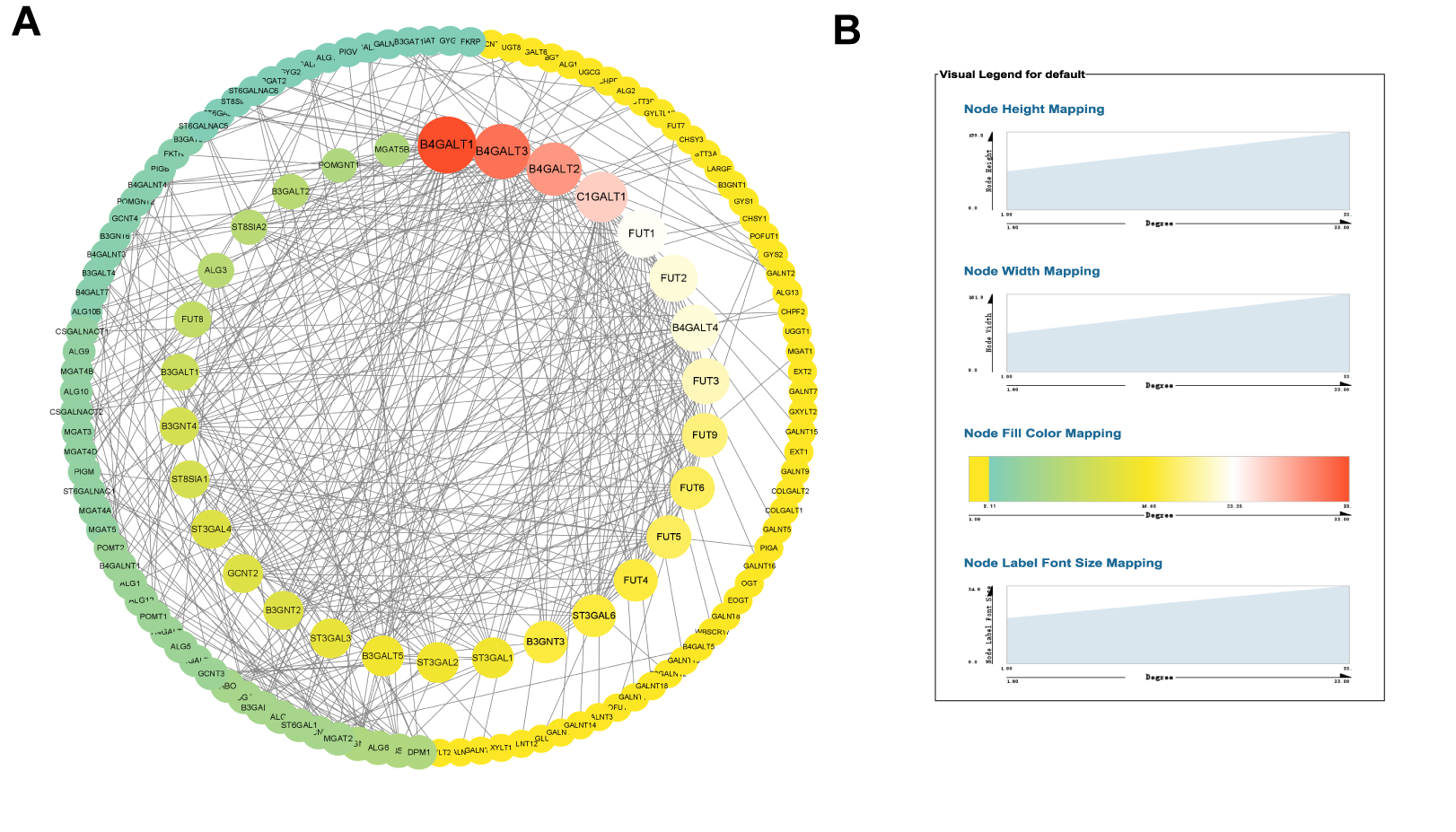

Supplement: Supplementary file 3 [file Image_2.tif]

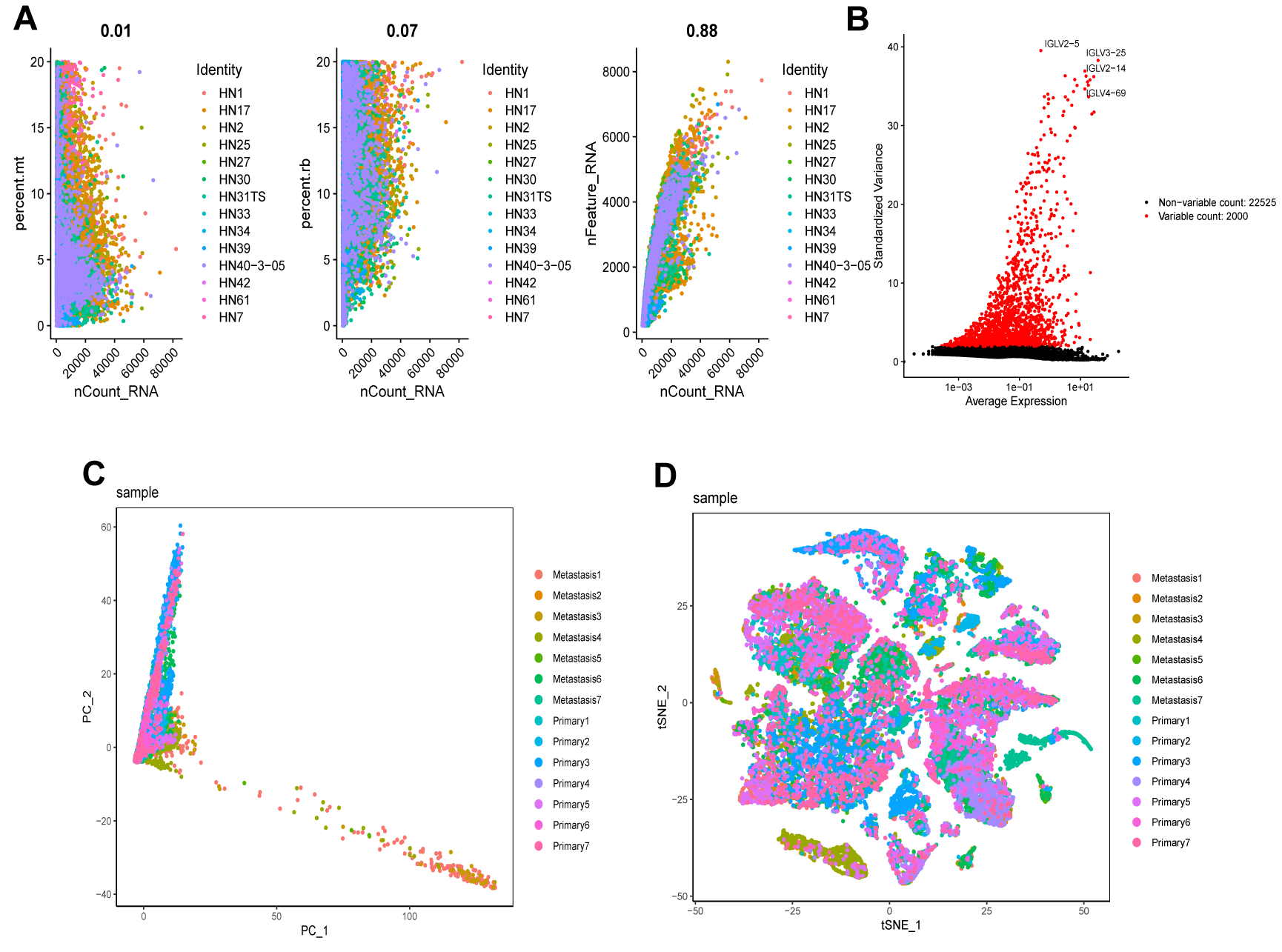

Supplement: Supplementary file 4 [file Image_3.tif]

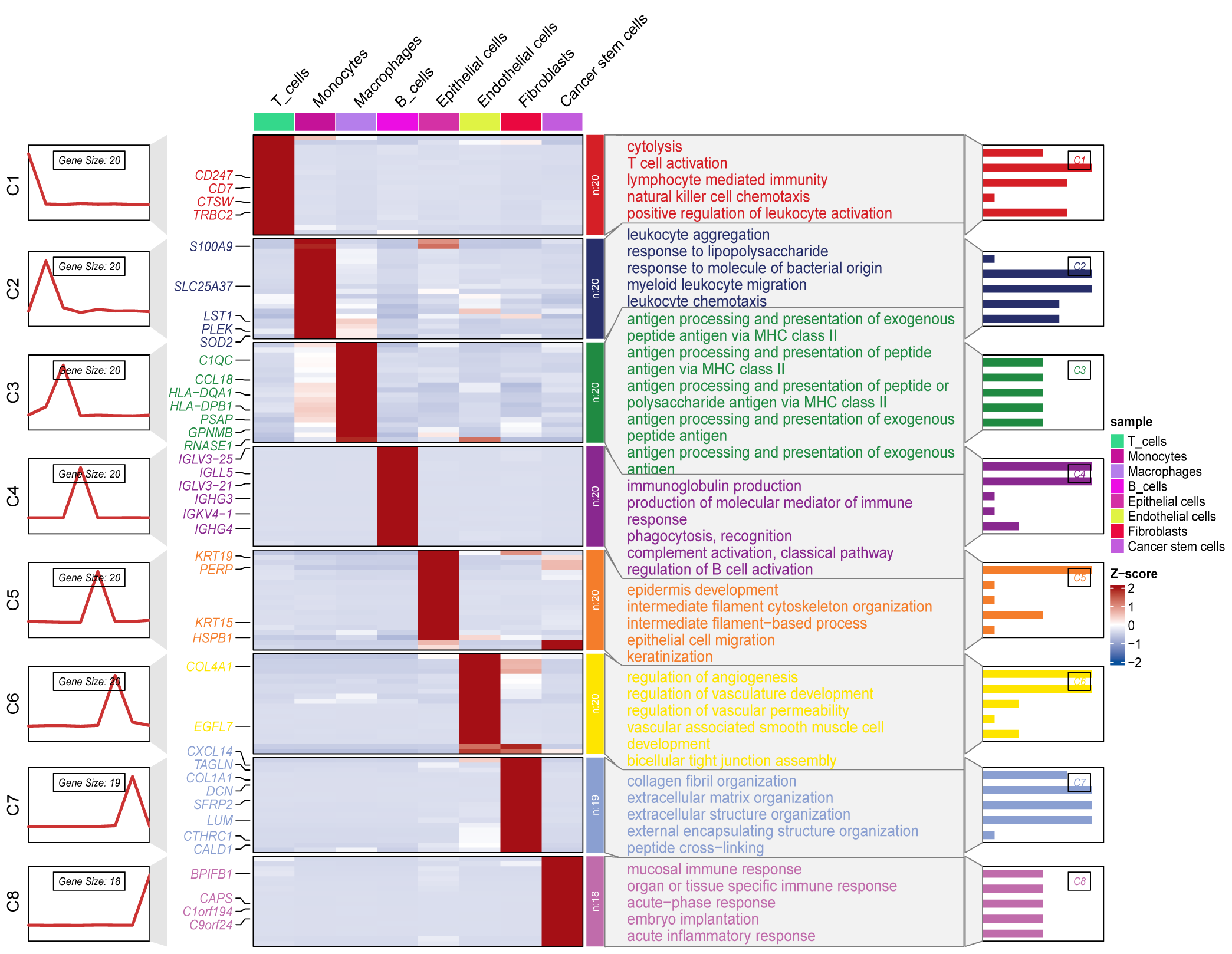

Supplement: Supplementary file 5 [file Image_4.tif]

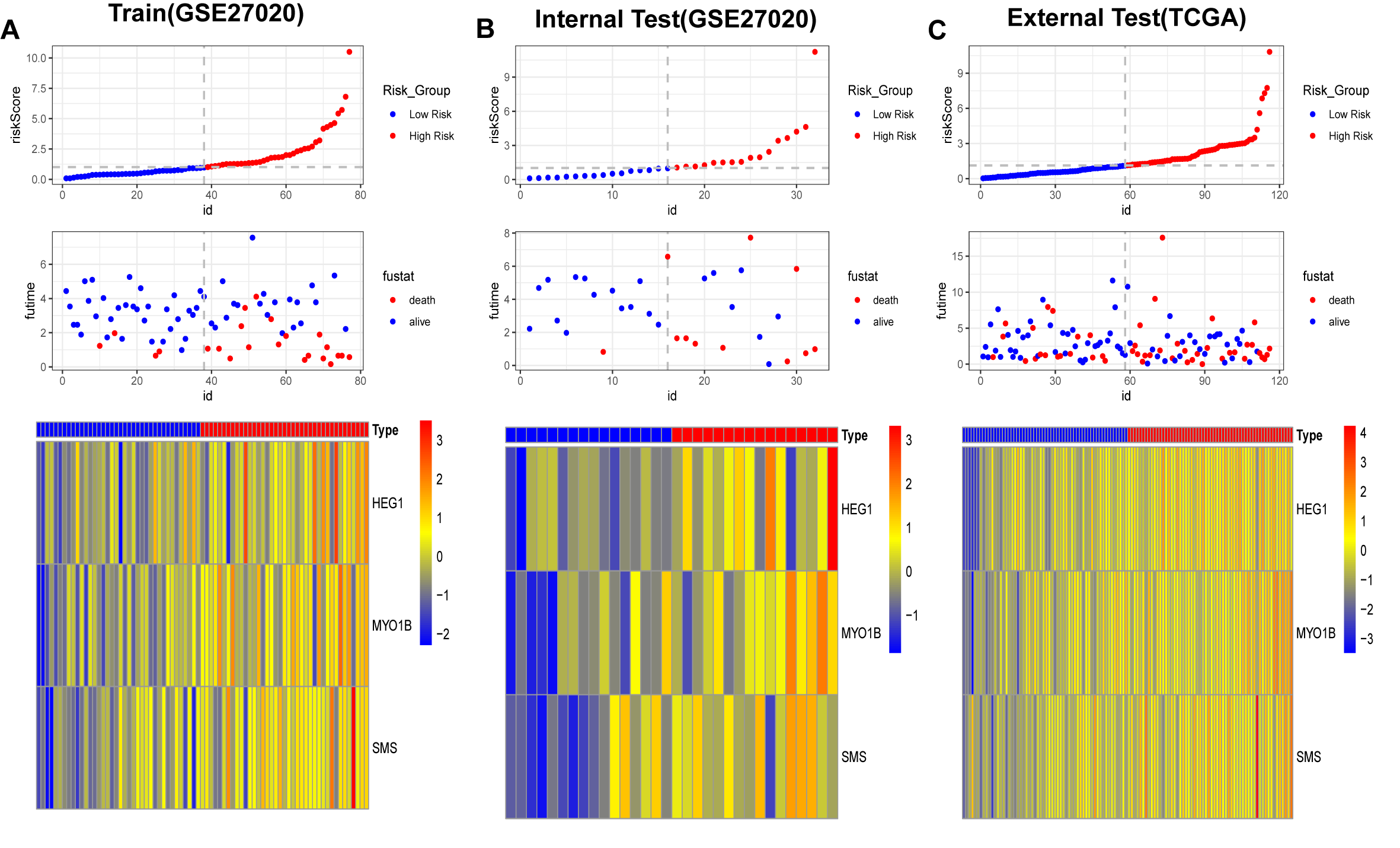

Supplement: Supplementary file 6 [file Image_5.tif]

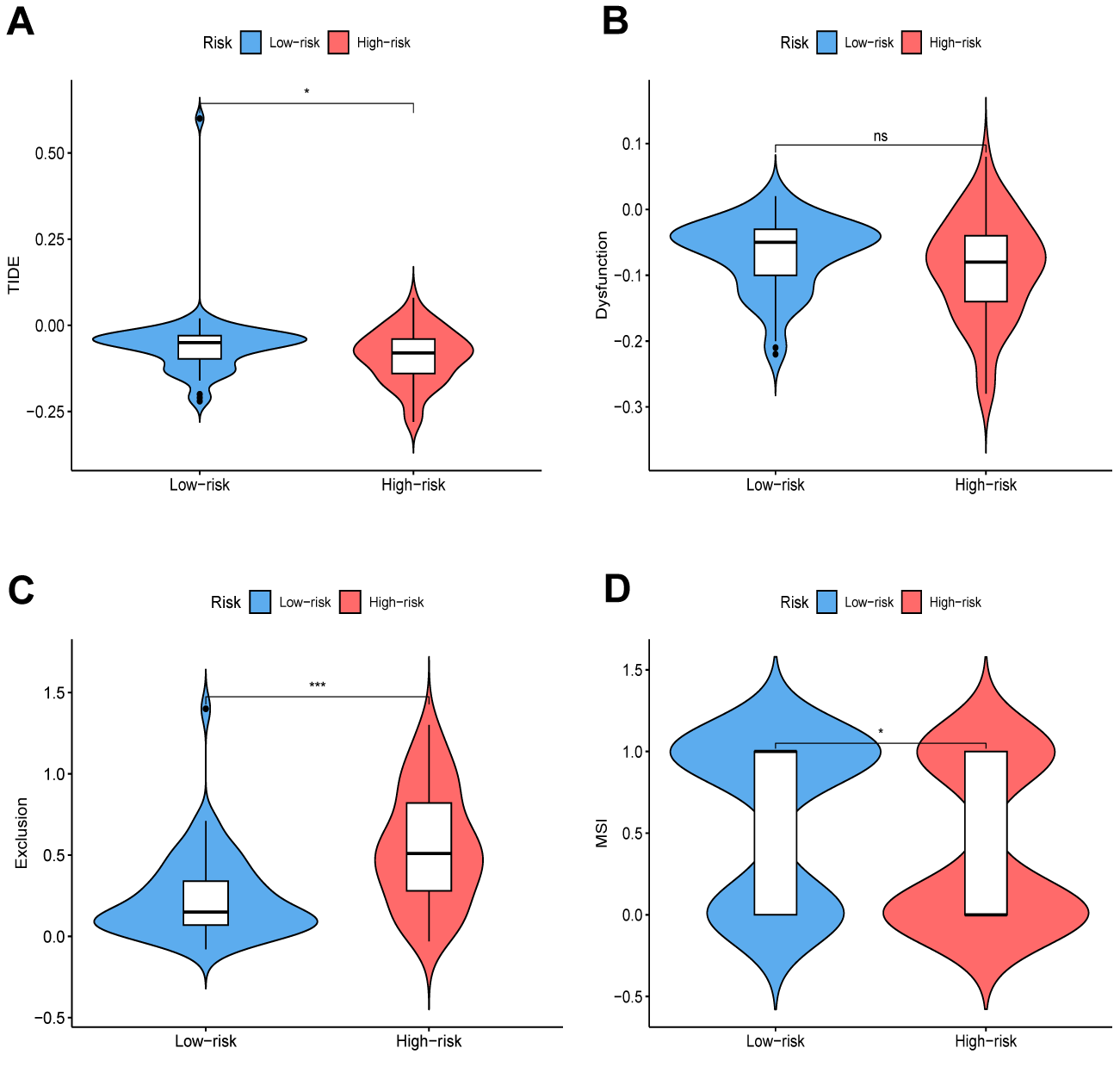

Supplement: Supplementary file 7 [file Image_6.tif]
