# Supplementary material for: Comprehensive investigation into the influence of glycosylation on head and neck squamous cell carcinoma and development of a prognostic model for risk assessment and anticipating immunotherapy
Source: Front Immunol. 2024 Mar 18;15:1364082. doi: 10.3389/fimmu.2024.1364082 (PMC10982401; doi:10.3389/fimmu.2024.1364082)
Supplement: Supplementary file 8 [file Table_1.docx]

|  | logFC | AveExpr | t | P.Value | adj.P.Val | B | abs.logFC |
| --- | --- | --- | --- | --- | --- | --- | --- |
| B3GNT6 | -1.913347529 | 4.233706546 | -11.15774704 | 1.48E-20 | 6.55E-18 | 36.15709722 | 1.913347529 |
| FUT6 | -1.606439287 | 5.185981368 | -11.72414618 | 5.96E-22  Results of differential analysis in scRNA. | 3.58E-19 | 39.31109103 | 1.606439287 |
| B3GALT5 | -1.354908542 | 4.091770002 | -6.80391519 | 3.62E-10 | 1.95E-08 | 12.86799468 | 1.354908542 |
| GCNT3 | -1.270501738 | 5.422943134 | -9.590634392 | 1.04E-16 | 2.33E-14 | 27.34257497 | 1.270501738 |
| GALNT5 | -1.030691965 | 5.392181779 | -7.978302839 | 7.62E-13 | 7.47E-11 | 18.64332519 | 1.030691965 |
| EXTL1 | -1.019104062 | 3.387794327 | -6.071024742 | 1.37E-08 | 5.10E-07 | 9.368592656 | 1.019104062 |
| ST6GALNAC1 | -0.999291086 | 5.858914398 | -8.06592392 | 4.75E-13 | 4.87E-11 | 18.85677069 | 0.999291086 |
| GALNT16 | -0.921454456 | 3.725803053 | -5.919426452 | 2.84E-08 | 9.69E-07 | 8.703279372 | 0.921454456 |
| ABO | -0.877350224 | 5.667194234 | -5.891096078 | 3.25E-08 | 1.09E-06 | 8.09555802 | 0.877350224 |
| FUT3 | -0.816623541 | 6.154702559 | -8.347378444 | 1.03E-13 | 1.21E-11 | 20.24155644 | 0.816623541 |
| GALNT12 | -0.768342541 | 5.762656207 | -9.207010254 | 8.91E-16 | 1.62E-13 | 25.21980602 | 0.768342541 |
| ST8SIA6 | -0.750919445 | 4.107698045 | -4.69647279 | 6.78E-06 | 0.000112063 | 3.568171187 | 0.750919445 |
| ST6GAL2 | -0.717845693 | 4.173770444 | -3.524449746 | 0.000591174 | 0.004870535 | -0.569430098 | 0.717845693 |
| B3GNT7 | -0.612680185 | 5.755400421 | -6.065356009 | 1.41E-08 | 5.21E-07 | 8.974712222 | 0.612680185 |
| GALNT17 | -0.601147368 | 4.459902674 | -3.97259437 | 0.000118572 | 0.001280928 | 0.860179921 | 0.601147368 |
| GYS2 | -0.566855639 | 3.007344813 | -3.260560585 | 0.00142842 | 0.009960891 | -1.358329687 | 0.566855639 |
| B3GNT3 | -0.543165111 | 6.255197794 | -6.137782377 | 9.93E-09 | 3.80E-07 | 8.997472161 | 0.543165111 |
| B3GNT8 | -0.491972078 | 5.950177488 | -5.528942717 | 1.76E-07 | 4.76E-06 | 6.448112972 | 0.491972078 |
| ST3GAL4 | -0.480096155 | 6.081023569 | -9.802793974 | 3.16E-17 | 7.75E-15 | 28.474243 | 0.480096155 |
| GCNT4 | -0.465780933 | 4.747132788 | -3.797824805 | 0.000225397 | 0.002192975 | 0.209619355 | 0.465780933 |
| XYLT1 | -0.463379439 | 5.385524344 | -3.356787939 | 0.001041343 | 0.007742694 | -1.491548963 | 0.463379439 |
| B3GALT1 | -0.40815304 | 3.298596069 | -2.376729539 | 0.01896388 | 0.078424949 | -3.571673402 | 0.40815304 |
| MGAT4A | -0.404778275 | 5.349753981 | -4.389346429 | 2.37E-05 | 0.000328498 | 2.103202275 | 0.404778275 |
| FUT2 | -0.402386822 | 6.276458062 | -4.816373377 | 4.10E-06 | 7.25E-05 | 3.202483313 | 0.402386822 |
| GCNT2 | -0.393347144 | 5.162150275 | -2.527892168 | 0.012704169 | 0.057255471 | -3.61397651 | 0.393347144 |
| GLT1D1 | -0.387042626 | 4.252060046 | -2.324900564 | 0.021667839 | 0.087056719 | -3.704865502 | 0.387042626 |
| B3GALT2 | -0.376931693 | 3.641320854 | -2.014046743 | 0.046122523 | 0.155495726 | -4.258652404 | 0.376931693 |
| TMTC2 | -0.372638638 | 5.695590705 | -4.13592342 | 6.39E-05 | 0.00076225 | 0.992559397 | 0.372638638 |
| MGAT3 | -0.346993118 | 5.067280684 | -1.993370868 | 0.048369043 | 0.161203921 | -4.698877708 | 0.346993118 |
| B3GALT4 | -0.317326196 | 6.160050385 | -5.017984013 | 1.73E-06 | 3.44E-05 | 4.180435559 | 0.317326196 |
| ALG11 | -0.299260473 | 4.618462901 | -3.827862931 | 0.000202123 | 0.002005273 | 0.390545148 | 0.299260473 |
| GALNT4 | -0.294848717 | 3.363674562 | -2.730331464 | 0.007228746 | 0.036915245 | -2.744398741 | 0.294848717 |
| ALG14 | -0.281846105 | 5.118793938 | -3.650998047 | 0.000380694 | 0.003381694 | -0.362577655 | 0.281846105 |
| GALNT7 | -0.271028527 | 6.099152953 | -3.360075127 | 0.001030041 | 0.007669749 | -1.840894477 | 0.271028527 |
| ST3GAL1 | -0.260476379 | 6.185483751 | -2.933853183 | 0.003975269 | 0.022752415 | -3.147656998 | 0.260476379 |
| C1GALT1 | -0.258057361 | 5.95959055 | -4.956288891 | 2.25E-06 | 4.32E-05 | 4.090371755 | 0.258057361 |
| GCNT1 | -0.256497293 | 5.332966552 | -2.105402379 | 0.03723079 | 0.132589093 | -4.578818638 | 0.256497293 |
| B4GALT6 | -0.252037376 | 5.016464875 | -1.709099186 | 0.089881081 | 0.254605226 | -5.143504051 | 0.252037376 |
| ST8SIA5 | -0.243066832 | 3.146567977 | -2.611750779 | 0.010095933 | 0.047955482 | -3.156996385 | 0.243066832 |
| B4GALT5 | -0.237761271 | 6.6916178 | -5.757822088 | 6.09E-08 | 1.88E-06 | 7.035911225 | 0.237761271 |
| ST6GALNAC3 | -0.216732539 | 4.034525158 | -1.814834086 | 0.071915123 | 0.216488527 | -4.61984376 | 0.216732539 |
| ST3GAL6 | -0.212968144 | 4.881994581 | -1.72859164 | 0.0863184 | 0.246997475 | -5.032545819 | 0.212968144 |
| PIGB | -0.198890577 | 5.439601221 | -3.019671887 | 0.003061019 | 0.0184959 | -2.400235229 | 0.198890577 |
| B3GALNT1 | -0.184996902 | 5.465841169 | -1.629607086 | 0.105668486 | 0.286149972 | -5.480363685 | 0.184996902 |
| UGCG | -0.176256975 | 6.5729514 | -4.668322622 | 7.62E-06 | 0.000124694 | 2.498600896 | 0.176256975 |
| ALG9 | -0.172239603 | 4.94439348 | -2.502970398 | 0.013587997 | 0.060332778 | -3.489465671 | 0.172239603 |
| ST6GALNAC6 | -0.164967019 | 3.278356747 | -2.421424768 | 0.016876461 | 0.071580718 | -3.557200887 | 0.164967019 |
| GALNT15 | -0.159673523 | 5.105323656 | -0.871129502 | 0.385331188 | 0.66098 | -6.204148007 | 0.159673523 |
| ST8SIA4 | -0.148891221 | 5.304835539 | -1.222017717 | 0.223969725 | 0.477072613 | -5.943687806 | 0.148891221 |
| B3GNT5 | -0.138965919 | 6.331454397 | -1.747196031 | 0.083026504 | 0.240261152 | -5.853027362 | 0.138965919 |
| TMTC4 | -0.120991497 | 5.731808667 | -1.418527143 | 0.158492209 | 0.379400805 | -5.923117635 | 0.120991497 |
| A4GALT | -0.117921642 | 6.690486155 | -1.778576143 | 0.077707711 | 0.228791921 | -6.107298533 | 0.117921642 |
| GYG2 | -0.11735818 | 4.979566502 | -0.719095808 | 0.473406177 | 0.736758122 | -6.242724853 | 0.11735818 |
| GALNT1 | -0.11646162 | 6.730686385 | -2.78284781 | 0.006213516 | 0.032708189 | -3.937149691 | 0.11646162 |
| FUT10 | -0.10228017 | 5.367947162 | -1.214124997 | 0.226958362 | 0.481626002 | -5.967090104 | 0.10228017 |
| DPY19L3 | -0.094993813 | 5.500211459 | -1.708847264 | 0.089927898 | 0.25471356 | -5.330301174 | 0.094993813 |
| PIGV | -0.093543383 | 5.83019581 | -1.636297478 | 0.104259223 | 0.283301913 | -5.645130251 | 0.093543383 |
| B3GNT2 | -0.091335455 | 6.493430886 | -2.497113935 | 0.013803506 | 0.061120702 | -4.416013612 | 0.091335455 |
| POMT1 | -0.087471762 | 5.752063504 | -1.57616958 | 0.117480559 | 0.308807378 | -5.687182616 | 0.087471762 |
| GALNT14 | -0.085325461 | 5.974873488 | -0.433131565 | 0.66565571 | 0.869175643 | -6.968516525 | 0.085325461 |
| GBGT1 | -0.084878 | 5.089091788 | -0.8217679 | 0.412752827 | 0.685541147 | -6.206373082 | 0.084878 |
| STT3B | -0.079792003 | 6.730887609 | -2.844332386 | 0.005190962 | 0.028288246 | -3.744510576 | 0.079792003 |
| ALG13 | -0.07756345 | 5.702627217 | -1.835174033 | 0.068825403 | 0.209662047 | -5.221493478 | 0.07756345 |
| ALG6 | -0.074341332 | 5.806751121 | -1.498604236 | 0.136463945 | 0.343014116 | -5.831811334 | 0.074341332 |
| PIGM | -0.066323932 | 5.793819239 | -1.211646572 | 0.227902746 | 0.482802527 | -6.20221651 | 0.066323932 |
| MGAT1 | -0.057466524 | 6.537859584 | -1.705055458 | 0.090634964 | 0.256264345 | -6.044737618 | 0.057466524 |
| B4GALT1 | -0.057107655 | 6.87062253 | -1.533506727 | 0.127644511 | 0.327852804 | -6.641228872 | 0.057107655 |
| UGT8 | -0.053947613 | 5.20609285 | -0.173406643 | 0.862608559 | 0.985414329 | -6.571803089 | 0.053947613 |
| B3GAT1 | -0.053894154 | 3.342909358 | -0.520970658 | 0.603296607 | 0.82930998 | -6.244198303 | 0.053894154 |
| GALNT3 | -0.053154783 | 6.529707519 | -0.852297515 | 0.395657294 | 0.670084665 | -7.114946401 | 0.053154783 |
| EOGT | -0.052118755 | 6.032086031 | -1.138894046 | 0.256896266 | 0.521773622 | -6.437671972 | 0.052118755 |
| FUT9 | -0.041020783 | 3.005702048 | -0.585416106 | 0.559309279 | 0.799654542 | -6.951884307 | 0.041020783 |
| GYS1 | -0.040982735 | 6.668541233 | -1.255680601 | 0.211542697 | 0.459747947 | -6.80926484 | 0.040982735 |
| ST8SIA3 | -0.037519876 | 2.961916676 | -0.447289743 | 0.655429662 | 0.862068308 | -7.434188673 | 0.037519876 |
| POMGNT2 | -0.034532806 | 6.100909238 | -0.461291126 | 0.645380742 | 0.855890161 | -7.015987134 | 0.034532806 |
| ALG12 | -0.025874705 | 5.730731564 | -0.420493517 | 0.674837336 | 0.874202161 | -6.78254022 | 0.025874705 |
| GCNT7 | -0.024853182 | 3.422583773 | -0.322475034 | 0.7476244 | 0.905876826 | -6.286625314 | 0.024853182 |
| GYG1 | -0.021461531 | 6.257018323 | -0.382924299 | 0.702417611 | 0.888831908 | -7.157587992 | 0.021461531 |
| GALNT11 | -0.021335599 | 5.910219038 | -0.271488132 | 0.78645751 | 0.932014726 | -6.946277464 | 0.021335599 |
| MGAT4D | -0.019907576 | 2.943657052 | -2.086727576 | 0.038917703 | 0.136997706 | -6.084486025 | 0.019907576 |
| B4GALT4 | -0.018427629 | 6.206068056 | -0.217207858 | 0.828395773 | 0.961899912 | -7.16696667 | 0.018427629 |
| ST6GAL1 | -0.017977779 | 5.918864274 | -0.123634858 | 0.901800324 | 0.999649934 | -6.9795201 | 0.017977779 |
| ALG10B | -0.008842144 | 5.057664923 | -0.096797702 | 0.923040009 | 0.999649934 | -6.487455397 | 0.008842144 |
| B3GAT2 | -0.006944897 | 3.334700406 | -0.088921973 | 0.929284335 | 0.999649934 | -6.411466502 | 0.006944897 |
| ALG5 | -0.006325616 | 6.395731421 | -0.179836994 | 0.857567863 | 0.981864315 | -7.315483974 | 0.006325616 |
| MGAT4B | -0.005501206 | 6.582988095 | -0.184412934 | 0.853984388 | 0.979343034 | -7.474555981 | 0.005501206 |
| GLT8D1 | -0.003201786 | 6.271126202 | -0.088639001 | 0.929508776 | 0.999649934 | -7.22664883 | 0.003201786 |
| MGAT4C | -0.002897321 | 3.124470634 | -0.033404747 | 0.973404395 | 0.999649934 | -6.734076355 | 0.002897321 |
| B3GNTL1 | 0.01280992 | 4.723119732 | 0.141886501 | 0.88739504 | 0.999649934 | -6.322036844 | 0.01280992 |
| FKTN | 0.018768557 | 5.251062061 | 0.210409919 | 0.833685781 | 0.965733518 | -6.557118634 | 0.018768557 |
| DPM1 | 0.02196226 | 6.747567052 | 0.816940694 | 0.415495892 | 0.687790296 | -7.289115386 | 0.02196226 |
| RFNG | 0.023304996 | 6.286240692 | 0.543183169 | 0.587958118 | 0.819701288 | -7.077871211 | 0.023304996 |
| ALG8 | 0.031503462 | 6.285738928 | 0.611587192 | 0.541906824 | 0.787882195 | -7.033040714 | 0.031503462 |
| PIGA | 0.03671827 | 5.858555956 | 0.623411706 | 0.534134746 | 0.782285725 | -6.72511054 | 0.03671827 |
| FUT5 | 0.038298522 | 2.981723383 | 1.757680319 | 0.081217199 | 0.236372717 | -6.893699408 | 0.038298522 |
| PIGZ | 0.038385344 | 5.641592765 | 0.346510268 | 0.729534109 | 0.900021248 | -6.72550624 | 0.038385344 |
| STT3A | 0.041152666 | 6.629967029 | 1.064686014 | 0.289043015 | 0.561724035 | -6.932852838 | 0.041152666 |
| B3GALNT2 | 0.041913768 | 5.86136865 | 0.920964108 | 0.358819543 | 0.635796816 | -6.495958613 | 0.041913768 |
| ALG2 | 0.043482677 | 6.450134906 | 1.437845805 | 0.152943298 | 0.370315546 | -6.313913189 | 0.043482677 |
| GLT6D1 | 0.044375295 | 2.977594622 | 2.898020434 | 0.004426498 | 0.024823395 | -4.484273416 | 0.044375295 |
| ST6GALNAC4 | 0.045999385 | 6.34438469 | 0.945939702 | 0.345980001 | 0.623765352 | -6.809277458 | 0.045999385 |
| FUT4 | 0.04840853 | 5.12101887 | 0.500620979 | 0.617506481 | 0.838625112 | -6.377097253 | 0.04840853 |
| B3GLCT | 0.050407358 | 5.837075871 | 0.669756863 | 0.504231654 | 0.760685086 | -6.674601131 | 0.050407358 |
| FUT11 | 0.05450364 | 6.048530139 | 0.971349257 | 0.333225249 | 0.610418201 | -6.564567597 | 0.05450364 |
| TMTC1 | 0.062753364 | 5.545575816 | 0.292705992 | 0.77022574 | 0.92002583 | -6.676296349 | 0.062753364 |
| OGT | 0.06362047 | 6.30399354 | 1.276233146 | 0.204207764 | 0.449266394 | -6.402962826 | 0.06362047 |
| ALG1 | 0.065019311 | 5.895734321 | 1.272186529 | 0.205636947 | 0.451269823 | -6.124993571 | 0.065019311 |
| GALNT8 | 0.065563703 | 3.362947993 | 0.421725735 | 0.673939936 | 0.873734207 | -6.357739771 | 0.065563703 |
| B4GAT1 | 0.070210821 | 6.2209426 | 1.328925701 | 0.186260928 | 0.423124164 | -6.269287405 | 0.070210821 |
| MGAT5 | 0.077100907 | 6.37294898 | 1.387800858 | 0.167633651 | 0.393953609 | -6.300397398 | 0.077100907 |
| DPY19L4 | 0.086261169 | 5.96668747 | 1.50478895 | 0.134867359 | 0.34028627 | -5.841493203 | 0.086261169 |
| B4GALNT2 | 0.087014647 | 4.06691113 | 0.306812015 | 0.759490161 | 0.91218945 | -6.133648368 | 0.087014647 |
| B4GALT7 | 0.088169051 | 6.123638545 | 1.621999395 | 0.107289505 | 0.289431812 | -5.764611184 | 0.088169051 |
| COLGALT2 | 0.096646576 | 4.416707955 | 0.313967534 | 0.754062086 | 0.90857135 | -6.162643345 | 0.096646576 |
| ALG10 | 0.097356649 | 5.206569585 | 1.058525574 | 0.291829954 | 0.565088911 | -5.973032572 | 0.097356649 |
| B4GALT2 | 0.0994246 | 6.547415268 | 2.76127379 | 0.006613734 | 0.034383199 | -3.66915738 | 0.0994246 |
| GTDC1 | 0.100006838 | 5.315185761 | 1.06106645 | 0.290678274 | 0.563666964 | -6.023571676 | 0.100006838 |
| FUT1 | 0.101108537 | 5.961859742 | 1.326338227 | 0.187113736 | 0.424423899 | -6.078917226 | 0.101108537 |
| CHSY1 | 0.101640953 | 6.384069588 | 2.045219352 | 0.042903846 | 0.147500271 | -5.188409346 | 0.101640953 |
| FUT7 | 0.117706859 | 4.511803686 | 0.588191285 | 0.557450987 | 0.798560091 | -6.061323846 | 0.117706859 |
| FUT8 | 0.128174342 | 5.756431487 | 1.017054304 | 0.311065933 | 0.586643242 | -6.29695433 | 0.128174342 |
| GALNT9 | 0.129991694 | 3.182018798 | 2.605636513 | 0.010268376 | 0.048568828 | -3.61689655 | 0.129991694 |
| UGGT1 | 0.134193643 | 6.414849101 | 3.55275158 | 0.000536265 | 0.004496133 | -1.222551523 | 0.134193643 |
| POMGNT1 | 0.136123007 | 6.395065667 | 3.369895667 | 0.000996959 | 0.007470214 | -1.786366969 | 0.136123007 |
| MGAT2 | 0.137942908 | 3.645314003 | 1.288047171 | 0.200077151 | 0.443308783 | -5.485416108 | 0.137942908 |
| B3GALT6 | 0.138189974 | 6.323165436 | 2.755026069 | 0.006733938 | 0.034892118 | -3.488894237 | 0.138189974 |
| FKRP | 0.144530484 | 5.855275286 | 2.831668584 | 0.005388055 | 0.029175027 | -2.986808869 | 0.144530484 |
| MFNG | 0.152380196 | 5.852444773 | 1.352380738 | 0.178662547 | 0.411622052 | -5.950687195 | 0.152380196 |
| ST6GALNAC2 | 0.156095649 | 6.011178688 | 1.384371618 | 0.16867826 | 0.39561303 | -6.004488325 | 0.156095649 |
| EXT2 | 0.158276193 | 6.357926277 | 2.982635869 | 0.003428756 | 0.02024649 | -2.887856666 | 0.158276193 |
| CHPF2 | 0.161004359 | 6.553416416 | 3.524769886 | 0.000590524 | 0.00486593 | -1.401286154 | 0.161004359 |
| B4GALT3 | 0.161452597 | 6.437677152 | 5.053985843 | 1.48E-06 | 3.01E-05 | 4.408106511 | 0.161452597 |
| ST3GAL5 | 0.165581419 | 4.825187565 | 0.992918705 | 0.322642199 | 0.599262268 | -5.843414328 | 0.165581419 |
| CSGALNACT1 | 0.174455867 | 5.702549897 | 1.667935882 | 0.097798318 | 0.270835577 | -5.387790143 | 0.174455867 |
| EXTL3 | 0.178586698 | 6.240358186 | 3.181706472 | 0.001841654 | 0.012221192 | -2.223493316 | 0.178586698 |
| UGGT2 | 0.180664213 | 5.534024448 | 2.374477288 | 0.019074869 | 0.07876883 | -3.924852558 | 0.180664213 |
| A4GNT | 0.186047082 | 3.231417861 | 2.448877424 | 0.015697906 | 0.067658795 | -3.985790198 | 0.186047082 |
| POFUT1 | 0.187104243 | 6.48301037 | 4.607021508 | 9.82E-06 | 0.000154695 | 2.564591914 | 0.187104243 |
| DPY19L2 | 0.189456665 | 3.808639658 | 1.343714752 | 0.181442298 | 0.416024065 | -5.370382036 | 0.189456665 |
| GALNT13 | 0.194640956 | 4.295943251 | 0.768923513 | 0.443369803 | 0.711358154 | -5.897936737 | 0.194640956 |
| TMTC3 | 0.198686321 | 6.404750471 | 3.354160939 | 0.001050458 | 0.00778859 | -1.804933077 | 0.198686321 |
| POMT2 | 0.20836441 | 5.695475379 | 3.825461911 | 0.000203896 | 0.002017474 | 0.141419859 | 0.20836441 |
| DPY19L1 | 0.20855601 | 6.137371532 | 2.556667661 | 0.011747987 | 0.053961016 | -3.823491537 | 0.20855601 |
| POGLUT1 | 0.215791133 | 5.893843492 | 3.838350633 | 0.00019455 | 0.001940499 | 0.092831719 | 0.215791133 |
| GALNT2 | 0.221696699 | 6.527052539 | 3.390813688 | 0.000929782 | 0.007065746 | -1.766757771 | 0.221696699 |
| HAS3 | 0.223387998 | 6.812305117 | 1.900852093 | 0.059593082 | 0.188284229 | -5.738632733 | 0.223387998 |
| POFUT2 | 0.223853179 | 5.814234035 | 3.045976537 | 0.002822326 | 0.017314173 | -2.34213628 | 0.223853179 |
| CHPF | 0.229105638 | 6.848638781 | 4.2039432 | 4.92E-05 | 0.000610536 | 0.756178019 | 0.229105638 |
| GXYLT2 | 0.240763682 | 5.203930643 | 1.12179558 | 0.264071035 | 0.53126204 | -5.847850268 | 0.240763682 |
| XXYLT1 | 0.24854343 | 6.31571684 | 3.753939986 | 0.00026402 | 0.002496403 | -0.422673302 | 0.24854343 |
| XYLT2 | 0.25453074 | 6.326147873 | 5.005915888 | 1.82E-06 | 3.59E-05 | 4.327606776 | 0.25453074 |
| B3GNT9 | 0.259812157 | 6.101925645 | 3.138882308 | 0.00211022 | 0.013630871 | -2.219762225 | 0.259812157 |
| B3GAT3 | 0.260527868 | 6.512624287 | 5.120571237 | 1.10E-06 | 2.34E-05 | 4.695160033 | 0.260527868 |
| B4GALNT3 | 0.262170409 | 6.416309592 | 2.024750769 | 0.044994833 | 0.152686555 | -5.153564475 | 0.262170409 |
| ALG3 | 0.268005955 | 6.684827306 | 6.568414995 | 1.19E-09 | 5.63E-08 | 11.24557006 | 0.268005955 |
| EXT1 | 0.284700153 | 6.562164626 | 5.326039157 | 4.43E-07 | 1.06E-05 | 5.559820623 | 0.284700153 |
| LFNG | 0.290868951 | 6.358440042 | 2.407638695 | 0.017497435 | 0.07371265 | -4.277548886 | 0.290868951 |
| EXTL2 | 0.291732261 | 5.781246645 | 3.590169961 | 0.000471022 | 0.004035672 | -0.647450495 | 0.291732261 |
| HAS2 | 0.30439834 | 5.434980995 | 1.437532673 | 0.153032031 | 0.370512711 | -5.540421129 | 0.30439834 |
| COLGALT1 | 0.307091189 | 6.822119779 | 7.55545965 | 7.30E-12 | 5.75E-10 | 16.18355609 | 0.307091189 |
| GALNT10 | 0.309607805 | 6.028659612 | 3.609785417 | 0.000439888 | 0.003807902 | -0.700897802 | 0.309607805 |
| GLT8D2 | 0.331352951 | 5.657704463 | 2.166483947 | 0.032145903 | 0.118120193 | -4.373864036 | 0.331352951 |
| MGAT5B | 0.33145629 | 3.943285189 | 1.81144798 | 0.072440485 | 0.217588975 | -4.6659432 | 0.33145629 |
| LARGE2 | 0.344328985 | 6.449306506 | 2.630387001 | 0.009586311 | 0.046091077 | -3.774906571 | 0.344328985 |
| ST8SIA1 | 0.352212728 | 4.344355783 | 1.991369687 | 0.048591335 | 0.161763334 | -4.29763643 | 0.352212728 |
| ST3GAL3 | 0.356775879 | 3.650345972 | 4.96721166 | 2.15E-06 | 4.15E-05 | 4.623376207 | 0.356775879 |
| ST3GAL2 | 0.372772896 | 5.583634347 | 3.576837042 | 0.000493359 | 0.004192457 | -0.574738811 | 0.372772896 |
| CSGALNACT2 | 0.374531103 | 6.211607032 | 4.732571588 | 5.83E-06 | 9.86E-05 | 3.328752536 | 0.374531103 |
| HAS1 | 0.389676 | 4.271523037 | 1.630759954 | 0.105424562 | 0.285658646 | -4.915084279 | 0.389676 |
| GALNT18 | 0.449322425 | 6.449219334 | 5.789871088 | 5.24E-08 | 1.65E-06 | 7.787410999 | 0.449322425 |
| CHSY3 | 0.60305664 | 4.902053503 | 2.983942776 | 0.003415115 | 0.020188008 | -2.064593223 | 0.60305664 |
| GALNT6 | 0.608383692 | 6.187044548 | 3.355304307 | 0.001046481 | 0.007772684 | -1.456785936 | 0.608383692 |
| ST6GALNAC5 | 0.63747095 | 4.364841302 | 3.845612912 | 0.000189464 | 0.001899338 | 0.546351627 | 0.63747095 |
| ST8SIA2 | 0.65294191 | 4.070603618 | 3.517167684 | 0.000606136 | 0.004971132 | -0.558198131 | 0.65294191 |
| B4GALNT4 | 0.678002912 | 5.569197864 | 2.467188201 | 0.014953108 | 0.06513842 | -3.540468198 | 0.678002912 |
| B3GNT4 | 0.689194965 | 4.982210429 | 3.264058621 | 0.001412264 | 0.009878371 | -1.280809496 | 0.689194965 |
| CERCAM | 0.786155155 | 6.30390908 | 6.193787772 | 7.57E-09 | 2.98E-07 | 9.850773722 | 0.786155155 |
| B4GALNT1 | 1.319047998 | 5.150319969 | 4.210513207 | 4.79E-05 | 0.000598173 | 1.800102494 | 1.319047998 |
