# Supplementary material for: Comprehensive investigation into the influence of glycosylation on head and neck squamous cell carcinoma and development of a prognostic model for risk assessment and anticipating immunotherapy
Source: Front Immunol. 2024 Mar 18;15:1364082. doi: 10.3389/fimmu.2024.1364082 (PMC10982401; doi:10.3389/fimmu.2024.1364082)
Supplement: Supplementary file 9 [file Table_2.docx]

| **Oligonucleotides** | **Nucleotide sequence (5'-3')** |
| --- | --- |
| **siRNA** |  |
| Scramble control | GCUUCGCGCCGUAGUCUUA |
| Si-SMS-1 | CCTCTCAATGAAAGTGTTGAA |
| Si-SMS-2 | GCGATGTCTTAGACAATCTTA |
|  |  |
| **Primer** |  |
| GAPDH | GGCCTCCAAGGAGTAAGACC (forward) |
|  | AGGGGAGATTCAGTGTGGTG (reverse) |
| SMS | AACGGCAGATTACCACCCAT (forward) |
|  | TCTTCTTTGCCACTGCCCAT (reverse) |
|  |  |

**Table S2. Oligonucleotides used in research**
